# An Exploratory Field Study on Egg Quality and Yolk Functional Properties in Commercial and Local Laying Hen Breeds Fed Whole Hemp Seed Under Agroecological Conditions

**DOI:** 10.3390/foods15173095

**Published:** 2026-08-31

**Authors:** Luca Marchetti, Niko Gioacchino Zeni, Luisa Zaniboni, Carlotta Giromini, Davide Lanzoni, Paola Antonia Corsetto, Angela Maria Rizzo, Lorenzo Colombini, Alice Giulia Dal Borgo, Giuseppe Gambazza, Raffaella Rebucci

**Affiliations:** 1 Department of Veterinary Medicine and Animal Sciences (DIVAS), University of Milan, Via dell’ Università 6, 26900 Lodi, Italy; luca.marchetti1@unimi.it (L.M.); niko.zeni@unimi.it (N.G.Z.); carlotta.giromini@unimi.it (C.G.); davide.lanzoni@uniroma5.it (D.L.); raffaella.rebucci@unimi.it (R.R.); 2 Institute for Food, Nutrition and Health, University of Reading, Reading RG6 6EU, UK; 3 Department of Human Science and Quality of Life Promotion, Università Telematica San Raffaele, 00166 Rome, Italy; 4 Department of Pharmacological and Biomolecular Sciences “Rodolfo Paoletti”, University of Milan, Via Balzaretti 9, 20133 Milan, Italy; paola.corsetto@unimi.it (P.A.C.); angelamaria.rizzo@unimi.it (A.M.R.); 5 Department of Cultural and Environmental Heritage, University of Milan, Via Noto 6, 20142 Milan, Italy; lorenzo.colombini@unimi.it (L.C.); alice.dalborgo@unimi.it (A.G.D.B.); giuseppe.gambazza@unimi.it (G.G.)

**Keywords:** agroecology, laying hens, hemp seed, egg quality, phenols, fatty acids, antioxidant capacity, local breeds, poultry nutrition, sustainable production

## Abstract

The present exploratory study evaluated egg quality and yolk functional properties in one commercial hybrid (Lohmann Brown^®^), two Italian (Livorno and Milanino) and a French (Marans) local laying hen breeds reared under agroecological conditions and fed a diet enriched with 20% whole hemp seed (HS). A total of 120 laying hens (30 per breed) were raised with pasture access and received the experimental diet for nine months. Egg physical traits, yolk composition, cholesterol content, fatty acid profile, nutritional indices, total phenolic content (TPC), and total antioxidant capacity (TAOC) were assessed at 0, 3, 6, and 9 months. Breed significantly influenced egg characteristics, including yolk and albumen proportions, yolk to albumen ratio, yolk pigmentation, and selected fatty acids (*p* < 0.05). The adopted agroecological management system increased polyunsaturated fatty acids, particularly omega-6, omega-3, reduced the n-6/n-3 ratio, improved atherogenic and thrombogenic indices, and enhanced the health promoting index, desirable to undesirable fatty acid ratio, and essential fatty acid content (*p* < 0.05). TPC and TAOC also increased significantly over time (*p* < 0.01), indicating enhanced antioxidant properties of the yolk. Overall, the adopted agroecological management characterized by HS enriched diet improved the nutritional and functional quality of eggs. These findings support alternative farming strategies that enhance egg value and promote the conservation of local poultry genetic resources.

## 1. Introduction

The global food system is currently facing major challenges driven by rapid population growth. Food production must increase substantially while simultaneously ensuring safety, quality, and environmental sustainability. Within this context, eggs are a cost effective and nutrient-dense food matrix, providing high biological value proteins, essential fatty acids, vitamins, and minerals in highly bioavailable forms. Moreover, eggs represent a strategic asset for global food security, as their consumption is not constrained by cultural or religious barriers [1,2].

At the same time, egg production must respond to increasing societal demands for improved animal welfare and more sustainable practices across the entire food supply chain. In the European Union, regulatory and policy efforts are promoting a transition toward alternative farming systems, with the progressive replacement of conventional and enriched cages planned by the end of the decade [3]. Growing consumer awareness has further intensified interest in alternative production systems perceived as more ethical and sustainable [4]. In this scenario, farming models grounded in agroecological principles are gaining attention for their potential to enhance sustainability in food production. Agroecology integrates ecological concepts into agricultural systems to improve biodiversity, resilience, productivity, and environmental performance [5]. It also promotes reduced reliance on external inputs, thereby lowering environmental impacts, and incorporates socio-economic dimensions by valuing local knowledge, farmer participation, and equitable food systems. Collectively, these principles support productive, environmentally sound, and socially responsible agricultural practices capable of adapting to global change [6,7]. In this respect, agroecological approaches, commonly adopted by smallholder farmers, may offer a valuable framework for integrating traditional production systems while contributing to meeting global protein requirements by 2050 [5].

However, modern egg production is predominantly based on commercial hybrid laying hens selected primarily for high productive performance [8]. Although effective in maximizing output, such selection strategies have been identified as a major driver of livestock biodiversity erosion [9,10]. The widespread replacement of traditional breeds with a limited number of highly specialized lines has progressively narrowed the poultry gene pool, increasing genetic uniformity within production systems. While this uniformity enhances efficiency under commercial conditions it may reduce adaptive capacity and increase vulnerability to environmental stressors and climate-related challenges.

Biodiversity is fundamental to sustainable livestock systems, as within species genetic variability enables populations to adapt to changing environmental conditions. Local breeds represent a valuable reservoir of adaptive traits, such as heat tolerance, improved immune response, and efficient use of diverse feed resources. Their broader immunogenetic variability may enhance disease resistance and robustness in extensive systems. Valorization of local breeds aligns with agroecological principles, strengthening system resilience while conserving genetic, ecological, and socio-economic resources [9,10,11].

Concurrently, the projected increase in global protein demand is expected to expand monogastric animal production, intensifying feed–food competition and accelerating the search for alternative, sustainable protein sources for animal nutrition [12,13]. Among potential alternative feed ingredients, hemp (*Cannabis sativa* L.) has emerged as a promising candidate due to its low requirements for water and pesticides and its ability to enhance nutrient and trace element uptake from deeper soil layers [14]. The use of hemp-based products, including seeds (HS), cake, oil, flour, and fiber in animal feed is administered by Regulation (EU) 2022/1104. Only varieties listed in the EU Common Catalogue of Agricultural Plant Species may be used, provided that their delta-9-tetrahydrocannabinol (THC) content does not exceed 0.20% on a dry matter basis [15]. Despite compositional variability among genotypes, hemp seed exhibits a highly favorable nutritional profile comparable to flaxseed, a widely used oilseed in the feed industry, with similar crude protein (23–25%), lipid (25–35%), fiber, and ash contents (5–6%) [16]. Overall, the combination of digestible proteins, essential fatty acids, and bioactive compounds positions hemp seeds as a promising functional ingredient for monogastric nutrition, with potential benefits for animal health and productivity [14,15,16]. Previous studies have also shown that HS and related co-products can positively modulate the nutritional and functional profile of eggs. These feed materials can bring appreciable modifications in the fatty acid profile of eggs, reducing the risk of exposure to chronic degenerative diseases [17,18]. More in detail, omega-3 polyunsaturated fatty acids (n-3 PUFA) contained in yolk have been associated with reduced cardiovascular risk and brain development [18]. Therefore, the strategic inclusion of HS in laying hen diets may represent an effective approach to enhance consumer intake of functional PUFAs through egg consumption.

Considering these premises, rearing laying hens within agroecological production systems combined with sustainable feeding strategies based on local resources may represent a valuable strategy for promoting a more sustainable approach to poultry nutrition, while enhancing the potential of eggs as functional foods. However, scientific literature currently lacks studies evaluating the quality of eggs produced under such integrated systems. Therefore, the present study aimed to assess egg quality traits and yolk functional properties in different laying hen breeds, one commercial hybrid (Lohmann Brown^®^) and three local breeds (Livorno, Marans, and Milanino), reared in a rational grazing system based on agroecological principles and fed a whole hemp seed enriched diet.

## 2. Materials and Methods

### 2.1. Experimental Design and Animal Housing

Based on the nature of the study, the authors determined that formal ethical review and approval were not required. The study involved routine husbandry practices and a non-invasive dietary intervention using an authorized feed ingredient. No procedures involving pain, suffering, distress, blood sampling, tissue collection, euthanasia, or other invasive interventions were performed. Eggs were collected during normal production without additional manipulation of the animals. All hens were managed in accordance with national legislation and Council Directive 1999/74/EC concerning the welfare of laying hens. The present trial was performed within the Happy Hens project and joined the framework of a social chicken coop project launched in 2023 by the association Soulfood Forestfarms Hub Italia. The project was carried out in a regenerative agroforestry system located between the Corvetto and Vigentino neighborhoods, planted on land owned by the City of Milan in the Vettabbia Park and assigned under agrarian lease to the farm Società Agricola CasciNet (Milan, Italy).

One-hundred and twenty laying hens of the following breeds (30 laying hens/breed) were compared: Lohmann Brown^®^ (LOH), Livorno (LIV), Milanino (MIL) and Marans (MAR). As the Milanino is a relatively less known local breed, additional information on its origin and characteristics is provided in Supplementary Material File S1 [19,20,21]. All animals were vaccinated against Marek and Newcastle diseases. Beaks were not trimmed and no other pharmacological treatments were given.

All the animals were raised at CasciNet farm. In September 2024, at 52 weeks of age laying hens were transferred to dedicated mobile hen houses. A period of adaptation corresponding to one month (month 0) was guaranteed to the animals. Trial started in October and lasted 9 months, until the end of June. One mobile hen house (6 birds/m^2^) for each breed (4 in total) was allocated to farm pasture according to routine farm practices. Each hen house was equipped with 5 nests in compliance with European legislation (Council Directive 1999/74/EC). The pastureland on which the trial was conducted was not treated with pesticides and herbicides and comprised a variety of plant species at different physiological stages of development, including *Lolium perenne*, *Trifolium pratense* and *Triticum aestivum*. Concerning environmental conditions, natural temperature, humidity and photoperiod were guaranteed for the animals.

Feed and water were provided with manual feeders and automatic drinkers and were available ad libitum. During the adaptation period a commercial basal diet was administered to laying hens in mash form. Starting from October 2024 (trial start) all laying hens were fed the same basal diet enriched with whole hemp seed (HS) (*Cannabis sativa* L., variety Carmagnola) provided by a company located in Cuneo, Italy (Roero Green Flower, Castagnito, Italy). HS were administered at 20% of the total amount of feed distributed. Hemp seeds were distributed in additional circular feeders. Hemp seeds were gathered from plants sown from May to October 2024 and harvested in October 2024, before trial start. Feed was purchased from a local company (Mangimi Monti s.r.l, Oltrona San Mamette, Italy). According to producer the utilized HS variety and batch were in compliance with Regulation (EU) 2022/1104 having a THC content lower than 0.20%. No additional analyses of THC content were performed.

### 2.2. Eggs Physical Parameters

At months 0, 3, 6 and 9 a total of 10 eggs per breed were collected from nests to perform evaluations on physical parameters. More in detail, shell thickness, and diameter and length of the egg were recorded using a micrometer (Digital Stainless Hardened, France), and the shape index was calculated by dividing the diameter by the length and multiplying the result by 100. Eggs were individually weighed (Sartorius Analytic A200S, Sartorius AG, Goettingen, Germany) as whole, yolk (separated with a yolk separation cup for cooking use) and the shell (membranes included), which was obtained after cleaning with demineralized water and drying in an oven at 45 °C for 12 h [22]. Albumen weight was calculated as the difference between whole egg weight and the sum of yolk and shell weight. Edible part (yolk + albumen), albumen, yolk, shell percentage and edible fraction percentage were calculated according to the whole egg weight. Yolk pigmentation was visually assessed by using the DSM YolkFan™ method (dsm-firmenich, Kaiseraugst, Switzerland). The evaluation was performed by the same blinded and trained observer under laboratory conditions, and the observer had no information regarding the group of eggs being evaluated.

**Figure 1:**
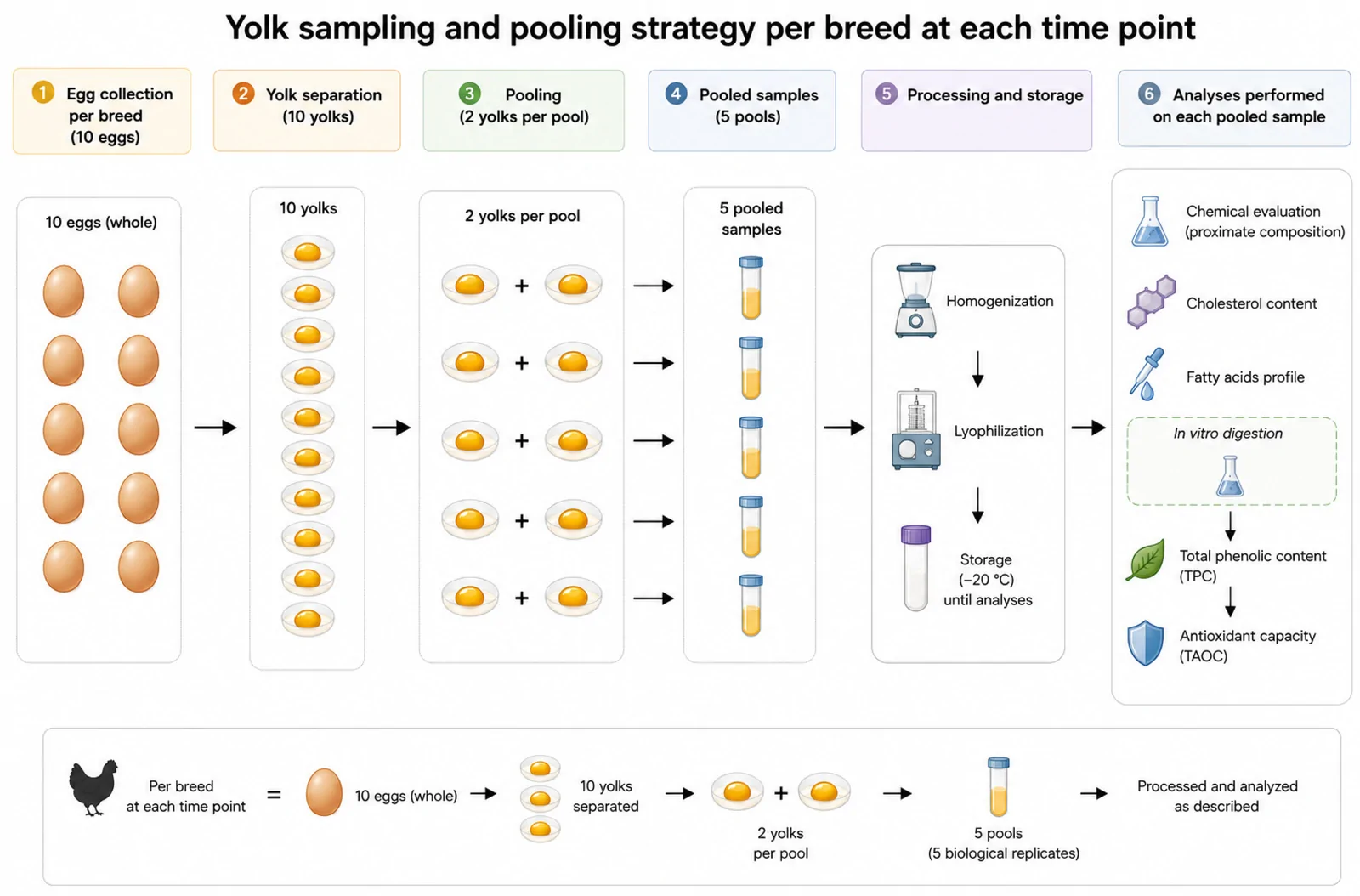
Yolk sampling and pooling strategy applied during the study. Created with the assistance of ChatGPT (GPT-5.6 Luna; OpenAI, San Francisco, CA, USA).

As resumed in Figure 1, following physical parameter evaluation 2 yolks from 10 eggs were pooled (5 replicates/breed), homogenized, lyophilized and stored until further analyses (chemical evaluation, cholesterol content, fatty acids profile, total phenolic content and antioxidant capacity).

### 2.3. Feed, Hemp Seeds and Yolk Chemical Evaluations

Feed, HS samples and lyophilized yolk (5 pooled replicates/breed) were analyzed for chemical composition, according to AOAC and AOCS methodologies [23,24]. In particular, dry matter (DM) was determined by drying the samples in a forced-air oven at 65 °C for 24 h (AOAC, 930.15). Ash content was measured following a 3 h incineration in a muffle furnace at 550 °C (AOAC, 942.05). Crude fiber (CF) was analyzed using the filtering bag method (AOCS, Ba 6a-05). Crude protein (CP) was determined by the Kjeldahl method considering the factor of 6.25 for nitrogen-to-protein conversion (AOAC, 2001.11). Ether extract (EE) was measured by Soxtec extraction with petroleum ether (AOAC, 2003.05). Basal diet and HS chemical analyses are listed in Table 1.

### 2.4. Assessment of Yolk Cholesterol Content and Fatty Acids Profile

Aliquots of lyophilized yolk (5 replicates/breed) collected at months 0, 3, 6 and 9 were used to determine cholesterol content and fatty acids profile. Cholesterol was determined using a commercial enzymatic photometric test-kit following the manufacturer’s instructions (R-biopharm, Darmstadt, Germany) and was expressed as g/100 g of yolk egg.

For yolk fatty acids characterization, lipids were extracted through the Folch method with minor modifications as reported in previous studies [25,26]. Freeze-dried yolk was homogenized through a chloroform/methanol 1:2 solution and lipids extract were recovered through centrifugation. Subsequently, two extractions were performed through chloroform/methanol, 2:1 and 1:1 (*v*/*v*), respectively. A total of 0.045 mM of 3,5-di-tert-4-butylhydroxytoluene (BHT) was contained in solvents used for extraction to avoid PUFA oxidation. Following lipids extraction, fatty acids determination was performed according to Marchetti et al. [26]. In particular, the fatty acids composition was assessed through a gas chromatographer (Shimadzu GC-2025, Shimadzu, Kyoto, Japan). Fatty acid methyl esters (FAMEs) were determined through lipid derivatization (sodium methoxide in methanol 3.33% (*w*/*v*)). C17:0 triglyceride was added to samples for correcting the reaction yield and recovery. Quantitative analysis was calibrated through a standard mixture (Sigma Aldrich, Milan, Italy) containing all fatty acid methyl esters.

Fatty acids content was assessed as fatty acids percentage: (fatty acid/total fatty acids) × 100. Afterwards, proportion of total saturated (SFA), monounsaturated (MUFA) and polyunsaturated fatty acids (PUFA) and the omega-6/omega-3 (n-6/n-3) ratio were calculated.

### 2.5. Estimated Enzyme Activity Evaluation

Estimated enzyme activity was determined according to Marchetti et al. [26] through specific FAMEs ratios to estimate the activity of desaturase ∆5 (20:4 n-6/20:3 n-6), ∆6 (18:3 n-6/18:2 n-6), stearoyl-CoA desaturase 16 (SCD-16, 16:1/16:0), stearoyl-CoA desaturase 18 (SCD-18, 18:1 n-9/18:0), elongase-5 (ELOVL-5, 20:3 n-6/18:3 n-6) and elongase-6 (ELOVL-6, 18:0/16:0).

### 2.6. Nutritional Value Indexes Calculation

Atherogenic (AI) and thrombogenic (TI) indices were calculated according to the equations proposed by Ulbricht and Southgate [27]. Desirable fatty acids (DFA; MUFA + PUFA + C18:0) and undesirable fatty acids (OFA; C12 + C14 + C16) were also determined, and their ratio was calculated. The nutritional value index (NVI; (C18:0 + C18:1)/C16:0) was computed following Daszkiewicz et al. [28]. Essential fatty acid (EFA) content was expressed as the sum of linoleic (C18:2), α-linolenic (C18:3α), and γ-linolenic (C18:3γ) acids. Additionally, the yolk health-promoting index (HPI) was calculated according to Goluch et al. [29].

### 2.7. Total Phenolic Content and Antioxidant Capacity of In Vitro Digested Yolk

Aliquots of lyophilized yolk (5 replicates/breed) collected at months 0, 3, 6 and 9 were used to determine total phenolic content (TPC) and antioxidant capacity (TAOC). Yolks were in vitro digested following the Infogest protocol [30,31]. Digested yolk samples were analyzed for their total phenolic content (TPC) through the Folin-Ciocalteu assay [32]. In addition, total antioxidant capacity (TAOC) of in vitro digested yolk was assessed using 2,2-azinobis-(3–ethyl-benzothiazoline-6–sulfonic acid (ABTS) assays according to Re et al. [33] with minor modifications as reported by Lanzoni et al. [34]. TPC results were expressed as mg/tannic acid equivalent (TAE)/100 g while ABTS results are reported as mg Trolox equivalent (TE)/100 g. A detailed methodology is provided in the Supplementary Materials (Supplementary File S2), with minor modifications from the protocol described by Lanzoni et al. [34].

### 2.8. Statistical Analysis

Statistical analyses were performed using GraphPad Prism 9.3.1 (GraphPad Software Inc., San Diego, CA, USA). Considering the exploratory nature of the present study and the lack of pen replication, for analyses of egg physical characteristics, individual eggs were considered the experimental unit (n = 10 per breed at each sampling time point), whereas for analyses performed on yolk samples, each pool consisting of two yolks was considered one biological replicate and represented the experimental unit (n = 5 per breed at each sampling time point).

Data were analyzed using a two-way ANOVA considering breed (LOH, LIV, MIL, and MAR), time (months 0, 3, 6, and 9), and their interaction (time × breed) as sources of variation. Since different eggs were collected at each sampling time, observations from different sampling times represented different experimental units. Following significant main effects or interactions, Tukey’s multiple-comparison test was used for appropriate pairwise comparisons. When the time × breed interaction was significant, comparisons among breeds within each sampling time and among sampling times within each breed were performed. When only the main effect of time was significant in the absence of a significant breed × time interaction, pairwise comparisons among sampling times were performed on the marginal means across breeds. Data were tested for normality using the Shapiro–Wilk test. Results are presented as mean ± standard error of the mean (SEM), and differences were considered statistically significant at *p* < 0.05. For pairwise comparisons, 95% confidence intervals (CI) were reported to quantify the precision and uncertainty of the estimated differences between means. Effect sizes were expressed as partial eta squared (ηp^2^), calculated as:

*ηp*^2^ = *SS_effect*/(*SS_effect* + *SS_error*)

where *SS_effect* is the Type III sum of squares associated with each main effect or interaction, and SS_error is the residual sum of squares.

The applied statistical model was the following:

*Y_ijk_* = *µ* + *Breed_j_* + *Time_k_* + (*Time* × *Breed*)*_jk_* + *e_ijk_*

where *Y_ijk_* is the *i*th recorded value for each parameter evaluated from eggs or pooled yolks belonging to *Breed_j_* (LOH, LIV, MIL, and MAR) at *Time_k_* (months 0, 3, 6, and 9), *μ* is the overall intercept, *Breed_j_* and *Time_k_* represent the fixed effects of breed and sampling time, respectively, (*Breed* × *Time*)*_jk_* represents their interaction, and *e_ijk_* represents the residual error.

## 3. Results

### 3.1. Eggs Physical Features

Results on egg physical parameters are reported in Table 2. Shell thickness was significantly affected only by time over the experimental period (*p* < 0.01, ηp^2^ = 0.47), whereas the shape index was exclusively influenced by breed (*p* < 0.01, ηp^2^ = 0.19). Whole egg weight was significantly affected by the breed × time interaction (*p* < 0.01, ηp^2^ = 0.23). Concerning differences among breeds within time points, at month 0, MIL hens produced heavier eggs than MAR (CI: 4.10–17.00, *p* < 0.01) and LIV hens (CI: 1.60–14.00, *p* < 0.01). At month 9, MAR eggs were heavier than LIV ones (CI: 1.20–13.00, *p* = 0.011). In terms of temporal changes, in MAR hens, egg weight increased from month 0 to month 3 (CI: 2.80–15.00, *p* < 0.01), month 6 (CI: 8.10–21.00, *p* < 0.01), and month 9 (CI: 6.30–19.00, *p* < 0.01). In LIV hens, egg weight increased from month 0 to month 3 (CI: 6.00–18.00, *p* < 0.01) and month 6 (CI: 1.20–13.00, *p* < 0.05). Egg weight at month 9 was lower than at month 3 (CI: −3.40 to −15.00, *p* < 0.01). In LOH hens, egg weight increased from month 0 to month 3 (CI: 3.60–15.00, *p* < 0.01) and month 6 (CI: 2.70 −14.00, *p* < 0.01). On the contrary egg weight at month 9 was lower than at month 3 (CI: −1.30 to −13.00, *p* < 0.01) and month 6 (CI: −0.32 to −12.00, *p* < 0.05).

Yolk percentage was significantly affected by breed (*p* < 0.01, ηp^2^ = 0.32) and by the breed × time interaction (*p* < 0.01, ηp^2^ = 0.22). At month 0, yolk percentage was higher in LIV and MIL hens than in MAR hens (CI: 2.30–8.30, *p* < 0.01; and CI: 3.60–9.40, *p* < 0.01), and LOH hens (CI: 2.00–7.60, *p* < 0.01; and CI: 3.20–8.70, *p* < 0.01). At month 6, MAR and MIL hens showed higher yolk percentages than LOH hens (CI: 1.00–6.50, *p* < 0.01; and CI: 1.20–6.80, *p* < 0.01). At month 9, yolk percentage remained higher in MAR (CI: 0.54–6.00, *p* = 0.012), LIV (CI: 0.50–6.00, *p* = 0.013), and MIL (CI: 2.80–8.30, *p* < 0.01) than in LOH.

Albumen percentage was significantly affected by breed (*p* < 0.01, ηp^2^ = 0.27) and by the breed × time interaction (*p* < 0.01, ηp^2^ = 0.19). At month 0, MAR hens showed higher albumen percentages than LIV (CI: 2.00–8.20, *p* < 0.01) and MIL hens (CI: 2.30–8.30, *p* < 0.01). Similarly, LOH hens showed higher values than LIV (CI: 2.30–8.20, *p* < 0.01) and MIL (CI: 2.60–8.30, *p* < 0.01). At month 6, LOH hens showed higher albumen percentages than MAR (CI: 0.58–6.30, *p* = 0.011) and MIL hens (CI: 0.88–6.70, *p* < 0.01). At month 9, LOH hens also showed higher albumen percentages than MAR (CI: 0.14–5.80, *p* = 0.036), LIV (CI: 0.02 −5.70, *p* = 0.047), and MIL hens (CI: 2.20–7.90, *p* < 0.01).

Shell and edible component percentages were influenced by time and breed (*p* < 0.01 ηp^2^ = 0.18 and *p* < 0.05 ηp^2^ = 0.08), without significant interaction between these factors. Shell percentage remained comparable between months 0 and 3 and between months 0 and 6, whereas it was significantly lower at month 9 than at month 0 (CI: −0.48 to −1.40; *p* < 0.01), month 3 (CI: −0.17 to −1.10; *p* = 0.003), and month 6 (CI: −0.12 to −1.00; *p* = 0.007). Conversely, edible percentage was higher at month 9 than at month 0 (CI: 0.48 to 1.40; *p* < 0.01), month 3 (CI: 0.17 to 1.10; *p* = 0.003), and month 6 (CI: 0.10 to 1.00; *p* = 0.009).

Yolk/albumen ratio was significantly affected by breed (*p* < 0.01, ηp^2^ = 0.28) and by the breed × time interaction (*p* < 0.01, ηp^2^ = 0.20), whereas the main effect of time was not significant. At month 0, MAR showed a lower ratio than LIV (CI: −0.20 to −0.05, *p* < 0.01) and MIL (CI: −0.22 to −0.07, *p* < 0.01). Similarly, LOH showed a lower ratio than LIV (CI: −0.19 to −0.05, *p* < 0.01) and MIL hens (CI: −0.21 to −0.07, *p* < 0.01). At month 6, MAR and MIL showed higher ratios than LOH (CI: 0.015–0.15, *p* = 0.01; and CI: 0.020–0.16, *p* = 0.0058). At month 9, MAR, LIV, and MIL showed higher ratios than LOH (CI: 0.0028–0.14, *p* = 0.038; CI: 0.0025–0.14, *p* = 0.030; and CI: 0.063–0.20, *p* < 0.01).

Yolk pigmentation was significantly affected by time (*p* < 0.01, ηp^2^ = 0.70) and breed (*p* < 0.01, ηp^2^ = 0.22), with a significant time × breed interaction (*p* < 0.01, ηp^2^ = 0.31). Concerning differences among breeds, at month 0, MAR pooled yolks showed lower pigmentation than LIV (CI: −1.90 to −0.04, *p* = 0.037), MIL (CI: −3.20 to −1.30, *p* < 0.01), and LOH (CI: −2.50 to −0.43, *p* = 0.002). At month 6, MAR and MIL hens had higher yolk pigmentation than LOH hens (CI: 0.35–2.10, *p* = 0.002, and CI: 0.23–2.00, *p* = 0.008). At month 9, MAR hens showed higher values than LIV (CI: 0.02–1.80, *p* = 0.043) and LOH (CI: 0.90–2.70, *p* < 0.01). Similarly, at month 9 MIL hens also evidenced improved pigmentation than LIV (CI: 0.30–2.10, *p* = 0.004) and LOH (CI: 1.20–2.90, *p* < 0.01). In MAR hens, yolk pigmentation increased from month 0 to month 3 (CI: 2.60–4.50, *p* < 0.01) and month 6 (CI: 3.80–5.70, *p* < 0.01); pigmentation also increased between months 3 and 6 (CI: 0.32–2.10, *p* = 0.003), whereas it decreased between months 6 and 9 (CI: −1.80 to −0.045, *p* = 0.035). In LIV, yolk pigmentation increased at month 3 (CI: 1.70–3.50, *p* < 0.01) and remained higher at month 6 (CI: 2.30–4.10, *p* < 0.01) and month 9 (CI: 1.10–2.90, *p* < 0.01) compared with month 0; a decrease was observed between months 6 and 9 (CI: −2.1 to −0.35, *p* = 0.002). In MIL, yolk pigmentation increased between months 0 and 3 (CI: 0.10 −2.00, *p* = 0.025), and month 6 (CI: 1.50–3.40, *p* < 0.01); significant differences were also observed between months 0 and 9 (CI: 0.94–2.80, *p* < 0.01) and months 3 and 6 (CI: 0.50–2.30, *p* < 0.01). In LOH hens, pigmentation increased from month 0 to month 3 (CI: 0.53 −2.50, *p* < 0.01) and month 6 (CI: 1.10–3.10, *p* < 0.01); the decrease from month 6 to 9 was significant (CI: −2.40 to −0.60, *p* < 0.01).

### 3.2. Yolk Chemical Evaluation

Results concerning yolk chemical composition are reported in Table 3. Dry matter (DM) was affected by breed (*p* < 0.01, ηp^2^ = 0.54), with no significant interaction between fixed effects. Crude protein (CP) was affected by time (*p* < 0.01, ηp^2^ = 0.22) and breed (*p* < 0.01, ηp^2^ = 0.42). Similarly, ether extract (EE) was influenced by both time (*p* < 0.01, ηp^2^ = 0.35) and breed (*p* < 0.01, ηp^2^ = 0.37), whereas the time × breed interaction was not significant. For both CP and EE no temporal changes were detected following Tukey’s test.

Ash content was significantly affected by time (*p* < 0.01, ηp^2^ = 0.50), breed (*p* < 0.01, ηp^2^ = 0.18), and their interaction (*p* < 0.01, ηp^2^ = 0.29). At month 0, MAR hens showed higher ash content than MIL (CI: 0.001–0.45, *p* = 0.049); the latter evidenced lower ash content than LIV (CI: −0.09 to −0.54, *p* = 0.002). At month 6, MAR, LIV, and MIL showed higher ash content than LOH (CI: 0.07–0.52, *p* = 0.006; CI: 0.09–0.550, *p* = 0.002; and CI: 0.017–0.47, *p* = 0.030). No significant differences among breeds were observed at months 3 or 9. In MAR hens, no significant differences in ash content were observed among different months. In LIV hens, ash content decreased from month 0 to month 3 (CI: −0.50 to −0.05, *p* = 0.011) and to month 6 (CI: −0.46 to −0.01, *p* = 0.036); ash content was higher at month 9 than at month 3 (CI: 0.58–0.13, *p* < 0.01) and month 6 (CI: 0.54–0.09, *p* = 0.003). In MIL hens, ash content was higher at month 9 than at month 0 (CI: 0.16–0.61, *p* < 0.01), month 3 (CI: 0.09–0.54, *p* = 0.003), and month 6 (CI: 0.16–0.61, *p* < 0.01). In LOH hens, ash content decreased from month 0 to month 6 (CI: 0.20–0.65, *p* < 0.01) and from month 3 to month 6 (CI: 0.11–0.54, *p* = 0.001), before increasing at month 9 (CI: 0.20–0.65, *p* < 0.01).

### 3.3. Yolk Cholesterol Content and Fatty Acids Composition

Yolk cholesterol content and fatty acids composition are reported in Table 4. Cholesterol concentration was exclusively affected by breed (*p* < 0.01, ηp^2^ = 0.23).

SFA content was significantly affected by time (*p* < 0.01, ηp^2^ = 0.72) and breed (*p* < 0.01, ηp^2^ = 0.33), with a significant time × breed interaction (*p* = 0.029, ηp^2^ = 0.25). Regarding differences among breeds within each time point, at month 0, MAR hens showed lower SFA content than LIV hens (CI: −4.80 to −0.53, *p* = 0.009), whereas LIV hens showed higher values than LOH hens (CI: 0.79–5.10, *p* = 0.003). At month 3, MAR hens showed higher SFA content than MIL hens (CI: 0.58–5.20, *p* = 0.008); LIV hens also showed higher values than MIL hens (CI: 0.22–4.5, *p* = 0.025). At month 6, MAR hens showed lower SFA content than LIV hens (CI: −4.30 to −0.02, *p* = 0.047). At month 9, MAR hens showed lower SFA content than LIV hens (CI: −4.9 to −0.63, *p* = 0.006), whereas LIV hens showed higher values than MIL hens (CI: 0.15–4.5, *p* = 0.032).

SFA longitudinally decreased in all four breeds during the study. In MAR hens, SFA content decreased from month 0 to month 6 (CI: −5.60 to −1.30, *p* < 0.01) and month 9 (CI: −5.80 to −1.50, *p* < 0.01), as well as from month 3 to month 6 (CI: −8.00 to −3.40, *p* < 0.01) and month 3 to month 9 (CI: −8.20 to −3.60, *p* < 0.01). In LIV hens, SFA content decreased from month 0 to month 6 (CI: −6.10 to −1.80, *p* < 0.01) and month 9 (CI: −5.70 to −1.40, *p* < 0.01), as well as from month 3 to month 6 (CI: −5.20 to −0.89, *p* = 0.002) and month 3 to month 9 (CI: −4.80 to −0.44, *p* = 0.012). In MIL hens, SFA content decreased from month 0 to month 6 (CI: −6.80 to −2.50, *p* < 0.01) and month 9 (CI: −6.50 to −2.20, *p* < 0.01), as well as from month 3 to month 6 (CI: −4.90 to −0.60, *p* = 0.007) and month 3 to month 9 (CI: −4.70 to −0.37, *p* = 0.016). In LOH hens, SFA content decreased from month 0 to month 6 (CI: −4.60 to −0.29, *p* = 0.020) and month 9 (CI: −4.50 to −0.20, *p* = 0.027), as well as from month 3 to month 6 (CI: −6.20 to −1.80, *p* < 0.01) and month 3 to month 9 (CI: −6.10 to −1.80, *p* < 0.01).

MUFA content was significantly affected by time (*p* < 0.01, ηp^2^ = 0.81) and breed (*p* < 0.01, ηp^2^ = 0.36), with a significant time × breed interaction (*p* = 0.013, ηp^2^ = 0.27). Breed-related differences were detected at month 0, when MAR showed higher MUFA content than LIV (CI: 3.70–13.00; *p* < 0.01), MIL (CI: 2.00–11.00; *p* = 0.002), and LOH (CI: 2.80–12.00; *p* < 0.01). At month 6, MAR showed higher MUFA content than LIV (CI: 2.90–12.00; *p* < 0.01), whereas LIV showed lower values than MIL (CI: −9.6 to −0.41; *p* = 0.028) and LOH (CI: −12 to −2.3; *p* = 0.001). No significant differences among breeds were detected at month 9. In MAR, MUFA concentrations decreased significantly from month 0 to month 6 (CI: −16.00 to −6.50; *p* < 0.01), and from month 0 to month 9 (CI: −21.00 to −12.00; *p* < 0.01). Further significant decreases were observed from month 3 to month 6 (CI: −11.00 to −0.70; *p* = 0.019), from month 3 to month 9 (CI: −16.00 to −6.10; *p* < 0.01) and from month 6 to month 9 (CI: −10.00 to −0.79; *p* = 0.015). In LIV, MUFA significantly decreased, with reductions observed between months 0 and 6 (CI: −15.00 to −5.70; *p* < 0.01), months 0 and 9 (CI: −17.00 to −7.50; *p* < 0.01), months 3 and 6 (CI: −16.00 to −7.20; *p* < 0.01), and months 3 and 9 (CI: −18.00 to −9.00; *p* < 0.01). In MIL significant decreases were observed from month 0 to month 6 (CI: −12.00 to −2.40; *p* < 0.01), from month 0 to month 9 (CI: −18.00 to −8.30; *p* < 0.01), from month 3 to month 6 (CI: −11.00 to −1.30; *p* = 0.007), from month 3 to month 9 (CI: −16.00 to −7.20; *p* < 0.01). In LOH, no significant differences were observed during the first part of the experimental period, whereas MUFA concentrations decreased significantly by month 9, with lower values than at month 0 (CI: −15.00 to −5.40; *p* < 0.01), month 3 (CI: −13.00 to −3.90; *p* < 0.01), and month 6 (CI: −10.00 to −1.10; *p* = 0.009).

PUFA, were conditioned by time (*p* < 0.01; ηp^2^ = 0.85), breed (*p* = 0.002; ηp^2^ = 0.20) and not by their interaction. In general, PUFA content increased significantly from month 0 to month 6 (CI: −15.00 to −9.10; *p* < 0.01) and from month 0 to month 9 (CI: −19.00 to −14.00; *p* < 0.01). Significant increases were also observed between months 3 and 6 (CI: −13.00 to −7.60; *p* < 0.01), months 3 and 9 (CI: −18.00 to −12.00; *p* < 0.01), and months 6 and 9 (CI: −7.30 to −1.80; *p* < 0.01).

The n-6 PUFA content was significantly affected by time (*p* < 0.01, ηp^2^ = 0.84), breed (*p* < 0.01, ηp^2^ = 0.27), and their interaction (*p* = 0.028, ηp^2^ = 0.25). Concerning differences among breeds, at month 0 MAR hens showed lower n-6 PUFA content than LIV (CI: −9.40 to −0.89, *p* = 0.012), MIL (CI: −9.10 to −0.62, *p* = 0.018), and LOH hens (CI: −11 to −2.9, *p* < 0.01). At month 3, MAR hens showed lower n-6 PUFA content than MIL hens (CI: −9.6 to −0.56, *p* = 0.022) and LOH hens (CI: −9.2 to −0.13, *p* = 0.041). At month 6, MAR hens showed lower n-6 PUFA content than LIV hens (CI: −8.60 to −0.09, *p* = 0.043). No differences among breeds were detected at month 9. Regarding time-dependent variations, in MAR hens n-6 PUFA content increased from month 0 to month 6 (CI: 7.00–16.00; *p* < 0.01) and month 9 (CI: 11.00–20.00; *p* < 0.01), as well as from month 3 to month 6 (CI: 5.20–14.00; *p* < 0.01) and month 9 (CI: 9.40–18.00; *p* < 0.01). In LIV hens, n-6 PUFA content increased from month 0 to month 6 (CI: 6.20–15.00; *p* < 0.01) and month 9 (CI: 7.40–16.00; *p* < 0.01), as well as from month 3 to month 6 (CI: 7.50–16.00; *p* < 0.01) and month 9 (CI: 8.70–17.00; *p* < 0.01). Similarly, in MIL hens, n-6 PUFA content increased from month 0 to month 6 (CI: 3.90–12.00; *p* < 0.01) and month 9 (CI: 7.80–16.00; *p* < 0.01), and from month 3 to month 6 (CI: 2.20–11.00; *p* < 0.01) and month 9 (CI: 6.00–15.00; *p* < 0.01). In LOH hens, significant increases were observed from month 0 to month 6 (CI: 0.26–8.80; *p* = 0.034) and month 9 (CI: 4.10–13.00; *p* < 0.01), as well as from month 3 to month 6 (CI: 1.20–9.70; *p* = 0.007) and month 9 (CI: 5.10–14.00; *p* < 0.01).

The n-3 PUFA content was exclusively affected by time (*p* < 0.01, ηp^2^ = 0.85). In general, n-3 PUFA content increased significantly from month 0 to month 3 (CI: 0.46–1.90, *p* < 0.01), with a further significant increase from month 3 to month 6 (CI: 1.40–2.80, *p* < 0.01) and from month 6 to month 9 (CI: 0.57–2.00, *p* < 0.01). The greatest overall difference was observed between months 0 and 9 (CI: 3.80–5.20, *p* < 0.01).

The n-6/n-3 PUFA ratio was significantly affected by time (*p* < 0.01, ηp^2^ = 0.87) and breed (*p* = 0.021, ηp^2^ = 0.14), whereas the breed × time interaction was not significant. Considering the marginal mean across breeds, the n-6/n-3 PUFA ratio decreased significantly from month 0 to month 3 (CI: −4.10 to −2.70, *p* < 0.01), from month 0 to month 6 (CI: −5.10 to −3.70, *p* < 0.01), and from month 0 to month 9 (CI: −5.70 to −4.30, *p* < 0.01). The ratio also decreased from month 3 to month 6 (CI: −1.70 to −0.30, *p* = 0.002) and from month 3 to month 9 (CI: −2.5 to −0.94, *p* < 0.01). Overall, the n-6/n-3 PUFA ratio showed a progressive decrease from month 0 to month 9, with the most pronounced changes occurring during the first six months of the experimental period.

Saturated and monounsaturated fatty acids characterized in pooled yolks are reported in Table 5. Palmitic (C16:0) content was significantly affected by time (*p* < 0.01, ηp^2^ = 0.79) and breed (*p* = 0.01, ηp^2^ = 0.28), whereas the time × breed interaction was not significant. In comparison to month 0, C16:0 content progressively decreased during the first six months, with lower values at month 3 (CI: −0.36 to −2.10; *p* = 0.0022), at month 6 (CI: −2.90 to −4.60; *p* < 0.01) and at month 9 (CI: −3.40 to −5.10; *p* < 0.01). Palmitic concentration in pooled yolks was also lower at month 6 than at month 3 (CI: −1.60 to −3.40; *p* < 0.01) and at month 9 than at month 3 (CI: −2.20 to −3.90; *p* < 0.01). Thus, across breeds the temporal pattern was characterized by a progressive decline in C16:0 yolk content from month 0 to month 6, followed by no further significant change at month 9.

Palmitoleic (C16:1) content was significantly affected by time (*p* < 0.01, ηp^2^ = 0.85) and breed (*p* < 0.01, ηp^2^ = 0.32), whereas the time × breed interaction was not significant. Across breeds, C16:1 content progressively decreased throughout the experimental period, with lower values at month 3 (CI: −0.33 to −0.82; *p* < 0.01), at month 6 (CI: −0.96 to −1.40; *p* < 0.01), and at month 9 (CI: −1.40 to −1.90; *p* < 0.01) in comparison to month 0. C16:1 content was also significantly lower at month 6 (CI: −0.38 to −0.88; *p* < 0.01) and at month 9 (CI: −0.80 to −1.30; *p* < 0.01) than at month 3. Finally, C16:1 content was further reduced between months 6 and 9 (CI: −0.18 to −0.67; *p* < 0.01). Thus, C16:1 showed a continuous decline from month 0 to month 9 in pooled yolks, with each successive months differing significantly from the preceding one.

Stearic (C18:0) content was significantly affected by time (*p* < 0.01, ηp^2^ = 0.51), breed (*p* < 0.01, ηp^2^ = 0.33), and their interaction (*p* < 0.01, ηp^2^ = 0.38). At month 0, C18:0 content was lower in MAR than in LIV (CI: −1.80 to −0.004; *p* = 0.048) and MIL (CI: −2.10 to −0.28; *p* = 0.006). At month 3, MAR and LIV showed higher C18:0 contents than MIL and LOH, with MAR differing from MIL (CI: −2.20 to −0.32; *p* = 0.005) and LOH (CI: −2.10 to −0.24; *p* = 0.009), and LIV differing from MIL (CI: −2.00 to −0.15; *p* = 0.016) and LOH (CI: −1.90 to −0.07; *p* = 0.030). At month 6, LIV showed higher C18:0 content than MAR (CI: 0.06–1.90; *p* = 0.032), MIL (CI: 0.55–2.40; *p* < 0.01), and LOH (CI: 0.57–2.40; *p* < 0.01). At month 9, MAR showed lower C18:0 content than LIV (CI: −1.80 to −0.01; *p* = 0.045), whereas no other significant breed differences were detected. Temporal comparisons within breeds showed that, in MAR hens, C18:0 increased from month 0 to month 3 (CI: 3.4 to 1.5; *p* < 0.01) and from month 0 to month 9 (CI: 1.90 to 0.06; *p* = 0.032). In LIV hens, C18:0 increased from month 0 to month 3 (CI: −2.2 to −0.40; *p* < 0.01) and from month 0 to month 9 (CI: −1.9 to −0.07; *p* = 0.029).

Oleic acid (C18:1) content was significantly affected by time (*p* < 0.01, ηp^2^ = 0.80), breed (*p* < 0.01, ηp^2^ = 0.36), and their interaction (*p* < 0.01, ηp^2^ = 0.29). At month 0, MAR hens showed higher C18:1 content than LIV (CI: 3.90 to 12.00; *p* < 0.01), MIL (CI: 2.10 to 11.00; *p* = 0.01), and LOH (CI: 3.0–11.00; *p* < 0.01). At month 6, LIV showed lower values than MIL (CI: −8.8 to −0.31; *p* = 0.031) and LOH (95% CI: −10 to −1.7; *p* = 0.002). By month 9, no significant differences among breeds were observed.

In MAR hens, C18:1 decreased from month 0 to month 3 (CI: −0.62 to −9.6; *p* = 0.019), month 6 (CI: −5.5 to −14; *p* < 0.01), and month 9 (CI: −11.00 to −19.00; *p* < 0.01). Similarly, it decreased from month 3 to month 6 (CI: −0.14 to −9.10; *p* = 0.041) and to month 9 (CI: −5.20 to −14.00; *p* < 0.01), and from month 6 to month 9 (CI: −0.85 to −9.30; *p* = 0.012). In LIV hens, C18:1 decreased from month 0 to month 6 (CI: −4.30 to −13.00; *p* < 0.01), from month 0 to month 9 (CI: −5.90 to −14.00; *p* < 0.01), from month 3 to month 6 (CI: −6.50 to −15.00; *p* < 0.01), and from month 3 to month 9 (CI: −8.00 to −16.00; *p* < 0.01). In MIL hens, C18:1 decreased from month 0 to month 6 (CI: −1.60 to −10.00; *p* = 0.003), from month 0 to month 9 (CI: −6.90 to −15.00; *p* < 0.01), from month 3 to month 6 (CI: −1.10 to −9.60; *p* = 0.008), from month 3 to month 9 (CI: −6.50 to −15.00; *p* < 0.01) and from month 6 to month 9 (CI: −1.10 to −9.60; *p* = 0.008). In LOH hens, C18:1 decreased from month 0 to month 9 (CI: −4.30 to −13.00; *p* < 0.01), from month 3 to month 9 (CI: −3.10 to −12.00; *p* < 0.01), and from month 6 to month 9 (CI: −0.81 to −9.30; *p* = 0.013). C18:1 generally decreased over the experimental period, particularly from month 3 onwards, although the magnitude and timing of these changes differed among breeds.

Polyunsaturated fatty acids contents characterized in pooled yolk are reported in Table 6. Linoleic acid (C18:2) content was significantly affected by time (*p* < 0.01, ηp^2^ = 0.85), breed (*p* < 0.01, ηp^2^ = 0.29), and their interaction (*p* < 0.05, ηp^2^ = 0.27). At month 0, MAR hens showed lower C18:2 content than LIV (CI: −1.40 to −9.80; *p* = 0.004), MIL (CI: −0.88 to −9.30; *p* = 0.011), and LOH (CI: −3.70 to −12.00; *p* < 0.01). At month 3, MAR showed lower values than MIL (CI: −0.54 to −9.40; *p* = 0.021) and LOH (CI: −0.59 to −9.50; *p* = 0.020). In terms of temporal changes, C18:2 evidenced the same trend in all four breeds, increasing throughout the trial. In MAR hens, C18:2 increased from month 0 to month 6 (CI: 7.60–16.00; *p* < 0.01) and month 9 (CI: 12.00–20.00; *p* < 0.01), as well as from month 3 to month 6 (CI: 5.50–14.00; *p* < 0.01) and month 9 (CI: 9.60–19.00; *p* < 0.01). Similarly, in LIV hens, C18:2 increased from month 0 to month 6 (CI: 6.20–15.00; *p* < 0.01) and month 9 (CI: 7.30–16.00; *p* < 0.01), and from month 3 to month 6 (CI: 7.50–16.00; *p* < 0.01) and month 9 (CI: 8.60–17.00; *p* < 0.01). In MIL hens, C18:2 increased from month 0 to month 6 (CI: 4.20–13.00; *p* < 0.01) and month 9 (CI: 7.90–16.00; *p* < 0.01), and from month 3 to month 6 (CI: 2.40–11.00; *p* < 0.01) and month 9 (CI: 6.10–14.00; *p* < 0.01). Finally, in LOH hens, C18:2 increased from month 0 to month 6 (CI: 0.33–8.70; *p* = 0.030) and month 9 (CI: 4.10–12.00; *p* < 0.01), and from month 3 to month 6 (CI: 1.30–9.70; *p* = 0.005) and month 9 (CI: 5.10–13.00; *p* < 0.01).

α-Linolenic acid (C18:3α) content was exclusively affected by time (*p* < 0.01, ηp^2^ = 0.84). Across breeds, C18:3α increased from month 0 to month 6 (CI: 1.90–3.20; *p* < 0.01) and month 9 (CI: 3.00–4.20; *p* < 0.01), from month 3 to month 6 (CI: 1.30–2.60; *p* < 0.01) and month 9 (CI: 2.40–3.60; *p* < 0.01), and from month 6 to month 9 (CI: 0.46–1.70; *p* < 0.01).

γ-linolenic acid (C18:3γ), content was significantly affected by time (*p* < 0.01, ηp^2^ = 0.67) and breed (*p* = 0.001, ηp^2^ = 0.22), whereas the time × breed interaction was not significant. Across breeds, C18:3γ increased progressively throughout the experimental period, from month 0 to month 3 (CI: 0.003–0.066; *p* = 0.028), at month 6 (CI: 0.05–0.11; *p* < 0.01), and at month 9 (CI: 0.09–0.16; *p* < 0.01). C18:3γ also increased from month 3 to month 6 (CI: 0.011–0.074; *p* = 0.004) and month 9 (CI: 0.062–0.13; *p* < 0.01), and from month 6 to month 9 (CI: 0.020–0.082; *p* < 0.01).

Arachidonic acid (C20:4) content was significantly affected by time (*p* < 0.01, ηp^2^ = 0.44), breed (*p* < 0.01, ηp^2^ = 0.55), and their interaction (*p* < 0.01, ηp^2^ = 0.35). Among breeds, at month 0 MAR hens showed higher C20:4 levels than LIV (CI: 0.28 to 0.78; *p* < 0.01) and LOH (CI: 0.43 to 0.93; *p* < 0.01). Similarly, MIL showed higher values than LIV (CI: 0.08–0.58; *p* = 0.004) and LOH (CI: 0.23 to 0.73; *p* < 0.01). At month 3, MAR hens showed higher C20:4 levels than LOH (CI: 0.09 to 0.62; *p* = 0.004) as in the case of MIL (CI: 0.16 to 0.66; *p* < 0.01). At month 6, MIL hens showed higher C20:4 levels than LOH (CI: 0.06 to 0.56; *p* < 0.01), and this difference was also observed at month 9 (CI: 0.07 to 0.56; *p* < 0.01). Over time, C20:4 decreased in MAR hens from month 0 to month 3 (CI: −0.059 to −0.062; *p* < 0.01), month 6 (CI: −0.91 to −0.40; *p* < 0.01), and month 9 (95% CI: −0.90 to −0.40; *p* < 0.01), and from month 3 to month 6 (95% CI: −0.60 to −0.07; *p* < 0.01) and month 9 (CI: −0.06 to −0.59; *p* < 0.01). In MIL hens, C20:4 decreased from month 0 to month 6 (CI: −0.01 to −0.51; *p* = 0.037) and month 9 (CI: −0.001 to −0.500; *p* = 0.048). No significant temporal differences were observed in LIV or LOH hens.

Regarding docosapentaenoic acid (C22:5) time (*p* = 0.001, ηp^2^ = 0.22) and time × breed interaction (*p* = 0.003, ηp^2^ = 0.32) were significant. At month 0, C22:5 was higher in MAR and LIV than in MIL hens (CI: 0.004–0.11; *p* = 0.026 and CI: 0.001–0.11; *p* = 0.042). At month 3, C22:5 was lower in LIV and MIL than in LOH (CI: −0.02 to −0.12; *p* = 0.005 and CI: −0.01 to −0.11; *p* = 0.010). In terms of temporal changes, no significant differences within breeds were detected following Tukey’s test.

### 3.4. Estimated Enzyme Activity Indices

**Figure 2:**
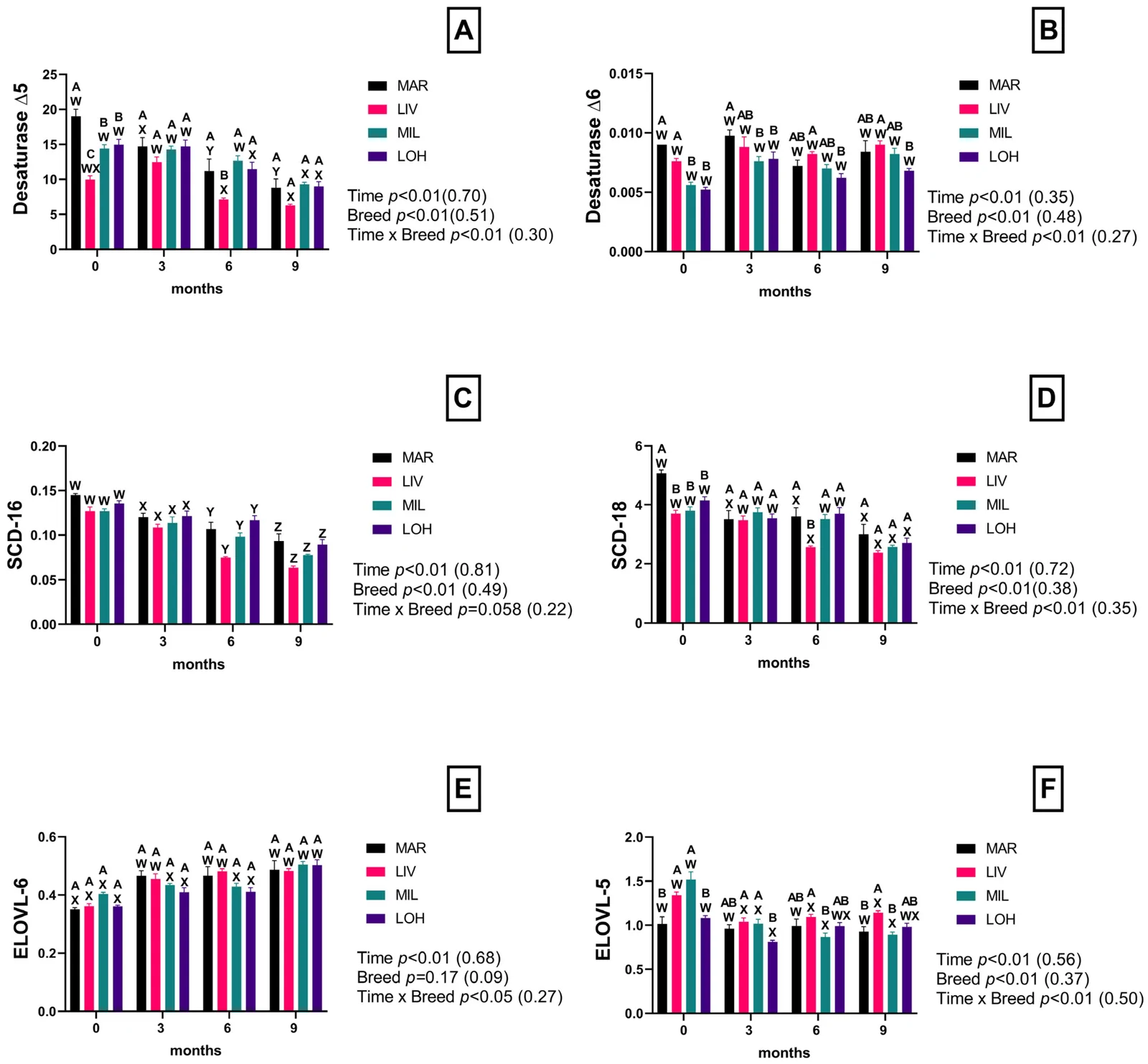
Desaturase ∆5 (**A**), desaturase ∆6 (**B**), stearoyl-CoA desaturase 16 (SCD-16, (**C**)), stearoyl-CoA desaturase 18 (SCD-18, (**D**)), elongase-6 (ELOVL-6, (**E**)) and elongase-5 (ELOVL-5, (**F**)) estimated during the study period. Abbreviations: MAR = Marans, LIV = Livorno, MIL = Milanino, LOH = Lohmann Brown^®^, SCD-16 = Stearoyl-CoA desaturase 16, SCD-18 = stearoyl-CoA desaturase 18, ELOVL-6 = elongase-6, ELOVL-5 = elongase-5. All the results are reported as mean ± standard error of the mean (SEM). Different letters indicate significant differences among breeds at the same sampling time (A, B, C: *p* < 0.05) and significant differences among sampling times within each breed (W, X, Y, Z *p* < 0.05). When only time was significant, temporal comparisons were performed on the marginal means of each month across breed and the same letters apply to all breeds (W, X: *p* < 0.05). Values sharing at least one superscript letter are not significantly different.

Estimated enzyme activity indices in yolk are reported in Figure 2. Δ5 desaturase activity was significantly affected by time (*p* < 0.01, ηp^2^ = 0.70) and breed (*p* < 0.01, ηp^2^ = 0.51) and their interaction (*p* = <0.01, ηp^2^ = 0.30; Figure 2A).

At month 0, MAR showed higher values than LIV (CI: 5.80–12.00; *p* < 0.01), MIL (CI: 1.40–7.80; *p* = 0.002), and LOH (CI: 0.85–7.20; *p* = 0.0073). LIV also differed from MIL (CI: −7.60 to −1.20; *p* = 0.003) and LOH (CI: −8.20 to −1.80; *p* < 0.01). At month 6, MAR Δ5 desaturase activity was higher than LIV (CI: 0.86–7.2; *p* = 0.007) which also differed from MIL (CI: −8.70 to −2.30; *p* < 0.01) and LOH (CI: −7.50 to −1.10; *p* = 0.004). In MAR, Δ5 desaturase activity decreased between months 0 and 3 (CI: −0.92 to −7.70; *p* = 0.007), 0 and 6 (CI: −4.60 to −11.00; *p* < 0.01), 0 and 9 (CI: −7.00 to −13.00; *p* < 0.01), 3 and 6 (CI: −0.16 to −6.90; *p* = 0.036), 3 and 9 (CI: −2.50 to −9.30; *p* < 0.01). In LIV, values decreased between months 3 and 6 (CI: −2.10 to −8.50; *p* < 0.01), 3 and 9 (CI: −3.00 to −9.40; *p* < 0.01). In MIL, significant decreases were observed between months 0 and 9 (CI: −2.00 to −8.30; *p* < 0.04), 3 and 9 (CI: −1.90 to −8.20; *p* < 0.01), and 6 and 9 (CI: −0.23 to −6.60; *p* = 0.031). In LOH, significant differences were observed between months 0 and 6 (CI: −0.34 to −6.70; *p* = 0.025), 0 and 9 (CI: −2.80 to −9.20; *p* < 0.01), 3 and 6 (CI: −0.09 to −6.40; *p* = 0.042), and 3 and 9 (CI: −2.50 to −8.90; *p* < 0.01). Overall, these results indicate that changes in Δ5 desaturase activity over time differed among breeds, with MAR exhibiting the most pronounced temporal variation.

Desaturase ∆6 was influenced by time (*p* < 0.01, ηp^2^ = 0.35) and breed (*p* < 0.01, ηp^2^ = 0.48) and Time × Breed interaction (*p* < 0.05, ηp^2^ = 0.27; Figure 2B). At month 0, MAR exhibited higher values than MIL (CI: 0.0017–0.0051, *p* < 0.01) and LOH (CI: 0.002–0.006, *p* < 0.01). Similarly, LIV showed higher values than MIL (CI: 0.0003–0.0040, *p* = 0.015) and LOH (CI: 0.0007–0.0041, *p* = 0.0024). At month 3, MAR showed higher values than MIL (CI: 0.0003–0.0040, *p* = 0.014) and LOH (CI: 0.0001–0.0038, *p* = 0.030). At month 6, LIV exhibited higher values than LOH (CI: 0.0003–0.004, *p* = 0.015). Similarly, at month 9, LIV showed higher values than LOH (CI: 0.0005–0.0039, *p* = 0.006). No relevant time-dependent changes were detected following Tukey’s test.

Stearoyl-CoA desaturase 16 (SCD-16) was significantly affected by time (*p* < 0.01, ηp^2^ = 0.81) and breed (*p* < 0.01, ηp^2^ = 0.49), whereas the time × breed interaction was not significant (Figure 2C). Hence, considering marginal means temporal changes, SCD-16 values progressively decreased throughout the experimental period. Values at month 0 were higher than those observed at month 3 (CI: 0.009–0.027, *p* < 0.01), month 6 (CI: 0.026–0.043, *p* < 0.01) and month 9 (CI: 0.044–0.062, *p* < 0.01). Furthermore, values at month 3 were higher than those at month 6 (CI: 0.008–0.026, *p* < 0.01) and month 9 (CI: 0.026–0.044, *p* < 0.01). Finally, month 6 values were higher than those at month 9 (CI: 0.009–0.027, *p* < 0.01).

Concerning stearoyl-CoA desaturase 18 (SCD-18) the effect of time was the largest, accounting for a substantial proportion of the variance (*p* < 0.01, ηp^2^ = 0.72), while breed also significantly influenced its values (*p* < 0.01, ηp^2^ = 0.38). A significant time × breed interaction was also detected (*p* < 0.01, ηp^2^ = 0.35, Figure 2D). At month 0, MAR evidenced higher SCD-18 values than LIV (CI: 0.71–2.00, *p* < 0.01), MIL (CI: 0.62–1.90, *p* < 0.01), and LOH (CI: 0.26–1.60, *p* = 0.003). At month 6, LIV had lower values than MAR (CI: −0.40 to −1.70, *p* < 0.01), MIL (CI: −1.6 to −0.31, *p* < 0.01) and LOH (CI: −1.80 to −0.49, *p* < 0.01). Over time, SCD-18 decreased in MAR hens from month 0 to month 3 (CI: −0.87 to −2.20; *p* < 0.01), month 6 (CI: −0.81 to −2.10; *p* < 0.01), and month 9 (CI: −1.40 to −2.70; *p* < 0.01). In LIV hens, SCD-18 decreased from month 0 to month 6 (CI: −0.50 to −1.80; *p* < 0.01) and month 9 (CI: −0.68 to −2.00; *p* < 0.01), while values at month 3 were higher than those at month 6 (CI: 0.26–1.6; *p* = 0.003) and month 9 (CI: 0.45–1.7; *p* < 0.01). In MIL hens, SCD-18 decreased from month 0 to month 9 (CI: −0.58 to −1.90; *p* < 0.01), from month 3 to month 9 (CI: −0.53 to −1.80; *p* < 0.01), and from month 6 to month 9 (CI: −0.30 to −1.60; *p* < 0.01). In LOH hens, SCD-18 decreased from month 0 to month 9 (CI: 0.80 to 2.1; *p* < 0.01) and from month 3 to month 9 (CI: 0.19 to 1.5; *p* = 0.006) and from month 6 to month 9 (CI: −0.19 to −1.50; *p* < 0.01).

Elongase-6 (ELOVL-6) was significantly affected by time (*p* < 0.01; ηp^2^ = 0.68), whereas breed did not significantly affect ELOVL-6 (*p* = 0.17; ηp^2^ = 0.09). The time × breed interaction was significant (*p* < 0.05; ηp^2^ = 0.27; Figure 2E). Following Tukey’s test, no relevant changes among breeds within time points were evidenced. Over time, ELOVL-6 increased in MAR hens from month 0 to month 3 (CI: 0.06–0.18; *p* < 0.01), month 6 (CI: 0.06–0.17; *p* < 0.01), and month 9 (CI: 0.08–0.19; *p* < 0.01). Similarly, ELOVL-6 increased in LIV hens from month 0 to month 3 (CI: 0.04–0.15; *p* < 0.01), month 6 (CI: 0.06–0.18; *p* < 0.01), and month 9 (CI: 0.07–0.18; *p* < 0.01). In MIL hens, ELOVL-6 increased from month 0 to month 9 (CI: 0.04–0.16; *p* < 0.01), as well as from month 3 to month 9 (CI: 0.01–0.13; *p* = 0.010) and from month 6 to month 9 (CI: 0.02–0.13; *p* = 0.0043). In LOH hens, ELOVL-6 increased from month 0 to month 9 (CI: 0.08–0.20; *p* < 0.01), month 3 to month 9 (CI: 0.04–0.15; *p* = 0.03), and month 6 to month 9 (CI: 0.04–0.15; *p* < 0.01).

Elongase-5 (ELOVL-5) activity was affected by time which effect was the largest, accounting for a substantial proportion of the variance (*p* < 0.01, ηp^2^ = 0.56), while breed also significantly influenced its values (*p* < 0.01, ηp^2^ = 0.37). A significant time × breed interaction was also detected (*p* < 0.01, ηp^2^ = 0.50, Figure 2F). At month 0, MAR evidenced lower ELOVL-5 values than LIV (CI: −0.14 to −0.51, *p* < 0.01) and MIL (CI: −0.32 to −0.69, *p* < 0.01), while LIV and MIL evidenced higher values than LOH (CI: 0.07 to 0.45, *p* < 0.01; CI: 0.25 to 0.62, *p* < 0.01). At month 3, both LIV and MIL showed higher ELOVL-5 values than LOH (CI: 0.042–0.41, *p* = 0.010; CI: 0.021–0.39, *p* = 0.024). At month 6, LIV evidenced higher ELOVL-5 values than MIL (CI: 0.041–0.41, *p* = 0.011). At month 9, LIV evidenced higher values than MAR (CI: −0.03 to −0.40, *p* = 0.019) and MIL (CI: 0.06–0.43, *p* = 0.0044). In LIV, ELOVL-5 values decreased from month 0 to month 3 (CI: −0.12 to −0.49; *p* < 0.01), month 6 (CI: −0.06 to −0.43; *p* < 0.01), and month 9 (CI: −0.02 t0 −0.39; *p* = 0.031). In MIL, ELOVL-5 values decreased from month 0 to month 3 (CI: −0.31 to −0.69; *p* < 0.01), month 6 (CI: −0.46 to −0.84; *p* < 0.01), and month 9 (CI: −0.44 to −0.81; *p* < 0.01). In LOH, ELOVL-5 values decreased from month 0 to month 3 (CI: −0.09 to −0.46; *p* < 0.01), while no significant changes were observed thereafter.

### 3.5. Nutritional Value Indices

**Figure 3:**
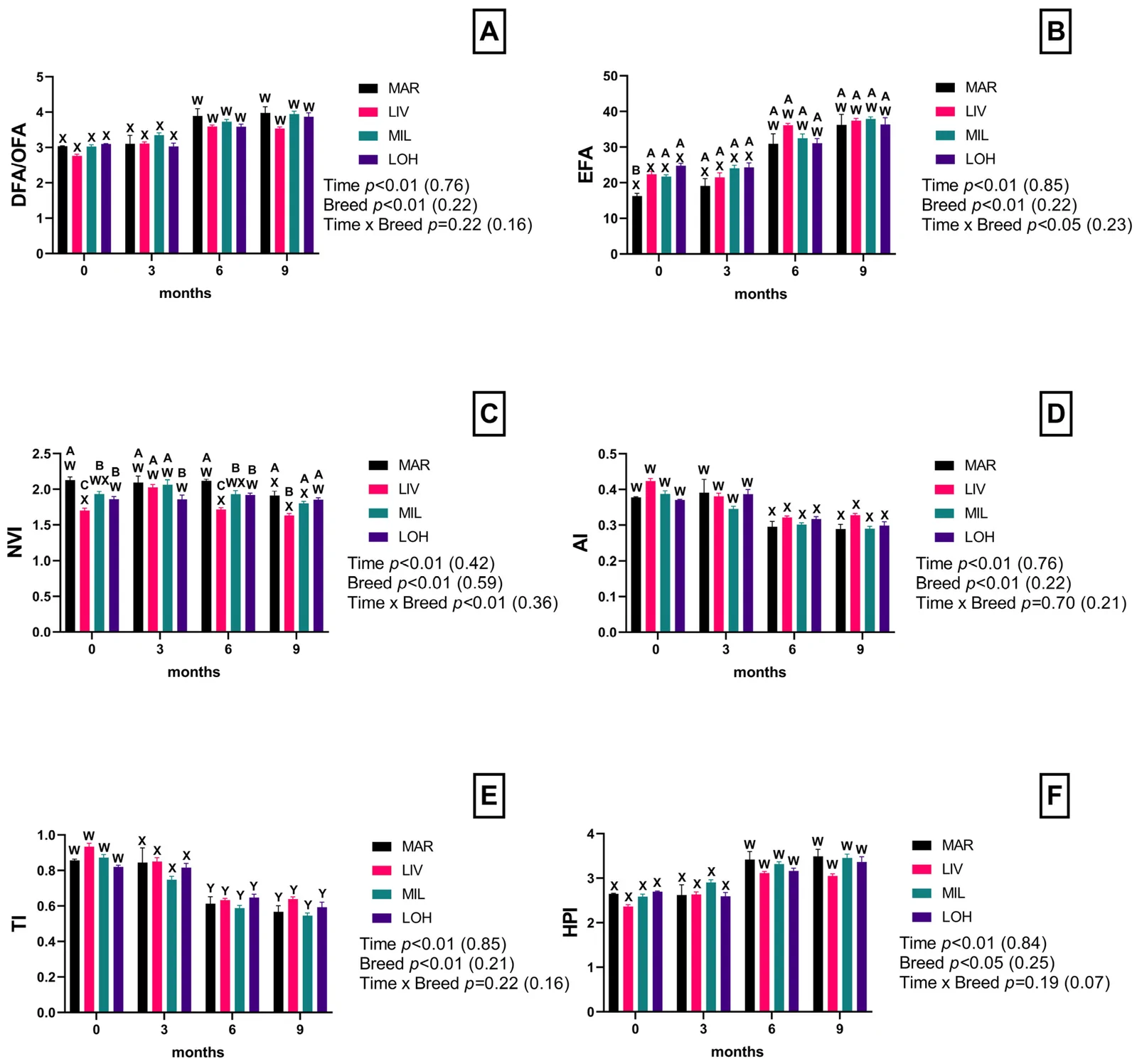
Desirable/undesirable fatty acids ratio (DFA/OFA, (**A**)), essential fatty acids (EFA, (**B**)), nutritional value index (NVI, (**C**)), atherogenic index (AI, (**D**)), thrombogenic index (TI, (**E**)) and health promoting index (HPI, (**F**)) evaluated during the study period. Abbreviations: MAR = Marans, LIV = Livorno, MIL = Milanino, LOH = Lohmann Brown^®^, DFA/OFA = desirable/undesirable fatty acids ratio, EFA = essential fatty acids, NVI = nutritional value index, AI = atherogenic index, TI = thrombogenic index, HPI = health promoting index. All the results are reported as mean ± standard error of the mean (SEM). Different letters indicate significant differences among breeds at the same sampling time (A, B, C: *p* < 0.05) and significant differences among sampling times within each breed (W, X, Y *p* < 0.05). When only time was significant, temporal comparisons were performed on the marginal means of each month across breed and the same letters apply to all breeds (W, X: *p* < 0.05). Values sharing at least one superscript letter are not significantly different.

Results concerning nutritional indices have been reported in Figure 3. The ratio among desirable and undesirable fatty acids compounds (DFA/OFA) was significantly affected by time (*p* < 0.01, ηp^2^ = 0.76) and breed (*p* < 0.01, ηp^2^ = 0.22), whereas the time × breed interaction was not significant (Figure 3A). Considering the marginal means across breeds, DFA/OFA values increased throughout the experimental period. Values at month 6 were higher than those observed at month 0 (CI: 0.53–0.91, *p* < 0.01) and month 3 (CI: 0.36–0.74, *p* < 0.01), while values at month 9 were higher than those at month 0 (CI: 0.66–1.00, *p* < 0.01) and month 3 (CI: 0.49–0.87, *p* < 0.01).

Yolks essential fatty acids (EFA) were influenced by time (*p* < 0.01, ηp^2^ = 0.85), breed (*p* < 0.01, ηp^2^ = 0.22), and their interaction (*p* < 0.05, ηp^2^ = 0.23; Figure 3B). At month 0, EFA values were lower in MAR than in LIV (CI: −0.74 to −11.00, *p* = 0.019), MIL (CI: −0.12 to −11.00, *p* = 0.043), and LOH (CI: −3.20 to −14.00, *p* < 0.01). No significant differences among breeds were observed at months 3, 6, or 9. Concerning temporal modulation of EFA, in MAR, values increased from month 0 to 6 (CI: −20 to −9.3; *p* < 0.01) and 9 (CI: −25 to −15; *p* < 0.01), as well as from month 3 to 6 (CI: −17 to −6.2; *p* < 0.01) and 9 (CI: −23 to −11; *p* < 0.01). In LIV, EFA values increased from month 0 to 6 (CI: −19 to −8.4; *p* < 0.01) and 9 (CI: −20 to −9.7; *p* < 0.01), and from month 3 to 6 (CI: −20 to −9.3; *p* < 0.01) and 9 (CI: −21 to −11; *p* < 0.01). In MIL, EFA values increased from month 0 to 6 (CI: −16 to −5.4; *p* < 0.01) and 9 (CI: −21 to −11; *p* < 0.01), as well as from month 3 to 6 (CI: −14 to −3.1; *p <* 0.01) and 9 (CI: −19 to −8.5; *p* < 0.01). In LOH, EFA values increased from month 0 to 6 (CI: −12 to −0.99; *p* = 0.014) and 9 (CI: −17 to −6.3; *p* < 0.01), and from month 3 to 6 (CI: −12 to −1.4; *p* = 0.007) and 9 (CI: −17 to −6.7; *p* < 0.01).

Nutritional value index (NVI) was influenced by time (*p* < 0.01, ηp^2^ = 0.42), breed (*p* < 0.01, ηp^2^ = 0.59), and their interaction (*p* < 0.01, ηp^2^ = 0.36; Figure 3C). At month 0, NVI values were higher in MAR than in LIV (CI: 0.26 to 0.59, *p* < 0.01), MIL (CI: 0.030 to 0.36, *p* = 0.014), and LOH (CI: 0.10 to 0.43, *p* < 0.01), while LIV showed lower values than MIL (CI: −0.40 to −0.068, *p* = 0.002) and LOH (CI: −0.04 to −0.60; *p* < 0.01). At month 3, MAR, LIV, and MIL showed higher NVI than LOH (CI: 0.061 to 0.41, *p* < 0.01; CI: 0.004 to 0.33, *p* = 0.043 and CI: 0.042 to 0.37, *p* = 0.008). At month 6, MAR showed higher NVI than LIV (CI: 0.24 to 0.57, *p* < 0.01), MIL (CI: 0.023 to 0.35, *p* = 0.019), and LOH (CI: 0.033 to 0.36, *p* = 0.013), while LIV showed lower values than MIL (CI: −0.05 to −0.40, *p* = 0.006) and LOH (CI: −0.04 to −0.40, *p* = 0.009). At month 9, LIV showed lower NVI values than MAR (CI: −0.12 to −0.45, *p* < 0.01), MIL (CI: −0.06 to −0.34, *p* = 0.039) and LOH (CI: −0.06 to −0.39, *p* = 0.004). In terms of temporal changes, NVI values remained unchanged across time in LOH. In MAR, NVI values decreased at month 9 compared with month 0 (CI: −0.052 to −0.38, *p* = 0.005), month 3 (CI: −0.008 to −0.360, *p* = 0.037), and month 6 (CI: −0.043 to −0.37, *p* = 0.008). In LIV, NVI values were higher at month 3 than at month 0 (CI: 0.49 to 0.16, *p* < 0.01) and subsequently decreased at month 6 (CI: −0.14 to −0.47, *p* < 0.01) and month 9 (CI: −0.23 to −0.56, *p* < 0.01). In MIL, NVI values were higher at month 3 than at month 9 (CI: 0.10 to 0.43, *p* = 0.04).

Atherogenic index (AI) was influenced by time (*p* < 0.01, ηp^2^ = 0.76) and breed (*p* < 0.01, ηp^2^ = 0.22), whereas the time × breed interaction was not significant (Figure 3D). Concerning temporal changes, IA values at month 0 were higher than at month 6 (CI: 0.061 to 0.10, *p* < 0.01) and month 9 (CI: 0.068 to 0.11, *p* < 0.01). Similarly, month 3 showed higher IA values than month 6 (CI: 0.046 to 0.088, *p* < 0.01) and month 9 (CI: 0.054 to 0.095, *p* < 0.01), while no difference was observed between months 6 and 9. Overall, AI decreased in yolks of all four breeds.

Similarly, thrombogenic index (TI) was influenced by time (*p* < 0.01, ηp^2^ = 0.85) and breed (*p* = 0.002, ηp^2^ = 0.21), whereas the time × breed interaction was not significant (Figure 3E). In terms of temporal variations, TI values were higher at month 0 than at month 3 (CI: 0.006 to 0.11, *p* = 0.021), month 6 (CI: 0.20 to 0.30, *p* < 0.01), and month 9 (CI: 0.24 to 0.33, *p* < 0.01). Month 3 also showed higher IT values than month 6 (CI: 0.14 to 0.24, *p* < 0.01) and month 9 (CI: 0.18 to 0.28, *p* < 0.01).

Finally, health-promoting index (HPI) was influenced by time (*p* < 0.01, ηp^2^ = 0.84) and breed (*p* = 0.04, ηp^2^ = 0.25; Figure 3F). HPI values were lower at month 0 than at month 6 (CI: −0.85 to −0.50, *p* < 0.01) and month 9 (CI: −0.94 to −0.59, *p* < 0.01), and at month 3 than at month 6 (CI: −0.74 to −0.39, *p* < 0.01) and month 9 (CI: −0.83 to −0.48, *p* < 0.01).

### 3.6. Total Phenolic Content and Antioxidant Capacity

**Figure 4:**
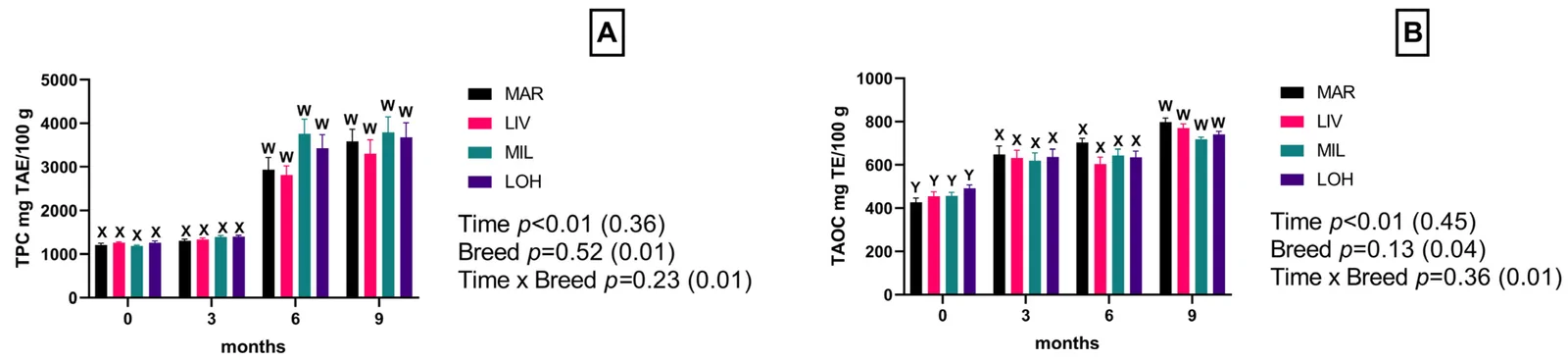
Total phenolic content (TPC, (**A**)) and total antioxidant capacity (TAOC, (**B**)) evaluated during the study period. Abbreviations: MAR = Marans, LIV = Livorno, MIL = Milanino, LOH = Lohmann Brown^®^, TPC = total phenolic content, TAOC = total antioxidant capacity. Values in parentheses represent partial eta squared (ηp^2^) reported as an effect size measure. All the results are reported as mean ± standard error of the mean (SEM). Since only time factor was significant, temporal comparisons were performed on the marginal means of each month across breed and the same letters apply to all breeds (W, X, Y: *p* < 0.05). Values sharing at least one superscript letter are not significantly different.

Total phenolic content (TPC) was significantly affected by time (*p* < 0.01, ηp^2^ = 0.36), whereas neither the main effect of breed nor the time × breed interaction was significant (Figure 4A). TPC increased significantly from month 0 to month 6 (CI: 1555 to 2452; *p* < 0.01) and month 9 (CI: 1910 to 2806; *p* < 0.01), and from month 3 to month 6 (CI: 1440 to 2310; *p* < 0.01) and month 9 (CI: 1795 to 2665; *p* < 0.01). No significant differences were observed between months 0 and 3 or between months 6 and 9.

Total antioxidant capacity (TAOC) values were exclusively affected by time (*p* < 0.01, ηp^2^ = 0.45; Figure 4B). TAOC increased from month 0 to month 3 (CI: 128 to 225; *p* < 0.01), month 6 (CI: 141 to 236; *p* < 0.01), and month 9 (CI: 251 to 348; *p* < 0.01). TAOC values also increased from month 3 to month 9 (CI: 75 to 171; *p* < 0.01) and from month 6 to month 9 (CI: 63 to 158; *p* < 0.01), whereas no significant difference was observed between months 3 and 6.

## 4. Discussion

The present exploratory field study evaluated the combined effects of breed and an agroecological rearing system with HS enriched diets on egg quality traits and yolk functional properties. Overall, the obtained results demonstrated that the integration of the applied agroecological management positively modulated yolk nutritional quality and functional potential in hens of different breeds.

More in detail, breed-related effects were evident in several physical egg quality traits. In all four breeds, shell and edible components percentages respectively decreased and increased throughout the trial. However, Marans, Milanino and Livorno hens exhibited lower albumen percentages, higher yolk percentages and yolk-to-albumen ratios compared with Lohmann hens at the end of the study. Egg composition is known to be strongly influenced by genetic background, with breeds differing in the relative deposition of lipids and proteins into yolk and albumen during follicular development. Local genotypes often produce eggs characterized by proportionally larger yolks, whereas highly selected commercial layers tend to allocate a greater proportion of egg mass to albumen as a consequence of long-term selection for egg number and laying efficiency [35,36,37]. Therefore, in accordance with the literature, the higher yolk proportion observed in Marans, Milanino and Livorno eggs may reflect breed-dependent characteristics related to different patterns of nutrient partitioning.

Breed-related differences were also observed in yolk pigmentation. Marans and Milanino eggs showed more intense yolk pigmentation than those of the other genotypes at the end of the study. Such variation may reflect breed-specific differences in foraging behavior, digestive capacity, carotenoid absorption and deposition. In addition, while Lohmann eggs showed comparable yolk pigmentation between months 0 and 9, Marans, Milanino, and Livorno hens showed a similar temporal pattern.

In general, access to pasture-based systems increases the availability of zeaxanthin, lutein, α-tocopherol and xanthophyll [38]. On the other hand, different authors underlined that pigments availability is not always constant in pasture-based systems due to seasonal variations, especially in comparison to caged systems in which hens can more easily uptake synthetic pigments through feed [39]. Nonetheless, hens can exhibit greater ranging activity and a higher capacity to exploit forage and other locally available feed resources in free-range and extensive systems. Fiorilla et al. [40] observed higher levels of grass-feeding and pecking behavior in the Robusta Maculata × Sasso breed compared with Lohmann Brown^®^ hens, which spent more time at the feeders when reared in free-range farming systems. Considering that in our study the four breeds were exposed to the same rearing conditions and dietary regime, it can be hypothesized that changes in yolk pigmentation may represent an indirect indicator of more effective pasture exploitation in local breeds throughout the study [40,41]. From a commercial perspective, darker yolks are often positively perceived by consumers and are frequently associated with natural production systems and superior egg quality [42]. Consequently, the greater yolk pigmentation observed in local breeds could contribute to increasing the market value of eggs produced under agroecological conditions.

The proximate composition of yolks highlighted the effects of breed and time on egg nutritional characteristics. Dry matter, crude proteins and ether extract levels fluctuated moderately but remained within the expected physiological range. These results are partially in agreement with Sirri et al. [43] who observed similar values of moisture, protein and lipids content in the yolk of conventional and local Italian breeds. Contrariwise to the latter study, ash content showed some breed-dependent variation over time. Nonetheless, these differences were not evident at the end of the study. This result is noteworthy, as it suggests that local breeds can produce eggs with nutritional qualities similar to those of commercial strains, supporting their commercial valorization [35].

Yolk cholesterol content was influenced by breed but remained unaffected by time and by the interaction between breed and time, indicating that the adopted agroecological rearing system did not significantly influence cholesterol deposition into the yolk of different breeds. This result is consistent with the concept that yolk cholesterol is under tight physiological regulation. Indeed, cholesterol represents an essential component for embryonic development and is therefore less responsive to dietary manipulation than fatty acid composition [44,45].

Interestingly, the progressive increase of PUFA levels in yolks from all four breeds observed during the nine-month experimental period suggested a cumulative beneficial effect of the adopted rearing conditions. Polyunsaturated fatty acids increased markedly throughout the experimental period, whereas saturated fatty acids declined. Simultaneously, the concentration of omega-3 PUFA increased in all breeds, resulting in a substantial reduction of the omega-6/omega-3 ratio. These findings were consistent with previous studies [17,18,46,47]. Hence, it can be hypothesized that the adoption of hemp-derived feed ingredients in the described agroecological conditions could effectively enhance the polyunsaturated fatty acid content of yolks, owing to their high levels of linoleic and α-linolenic acids, which also increased progressively over time in the present study.

The transfer of dietary PUFA into the yolk confirms the efficiency of the hen as a biological converter of dietary lipids into nutritionally valuable egg components, independently from the considered genotype. Indeed, although significant breed effects were detected for several lipid fractions at month 0, the time-dependent response to adopted agroecological rearing system with dietary HS enrichment was remarkably consistent among genotypes. Initial differences in SFA, MUFA and PUFA proportions progressively diminished throughout the study, suggesting that dietary lipid composition exerted a stronger influence on yolk fatty acid deposition than genetic background [35,48]. This observation is particularly relevant in agroecological systems, as it indicates that local breeds can efficiently produce nutritionally enhanced eggs when provided with appropriate feeding strategies.

The convergence observed in the major lipid fractions was mirrored by the profile of individual fatty acids. Although several Time × Breed interactions were detected throughout the study, these differences were generally transient and most evident during the intermediate phases of supplementation (months 3 and 6). By the end of the experimental period, the concentrations of linoleic (C18:2), α-linolenic (C18:3α) and γ-linolenic (C18:3γ) acids progressively increased in all four breeds, whereas the principal saturated and monounsaturated fatty acids declined. These findings suggest that the adopted agroecological rearing system with dietary HS enrichment progressively reduced the influence of genotype on the deposition of the major yolk fatty acid classes, leading to a common lipid phenotype across local and commercial genotypes. This result is particularly relevant from a consumer perspective, as deposition of essential fatty acids and omega-3 PUFA in yolks can contribute to the improvement of nutritional quality indices and enhances the functional value of eggs [49].

Arachidonic acid (C20:4) and docosapentaenoic acid (C22:5) are two long-chain PUFA generated through successive desaturation and elongation of linoleic and α-linolenic acids, respectively [50]. Although both precursors progressively increased and reached comparable concentrations among breeds by month 9, Milanino, Marans and Livorno hens generally exhibited higher concentrations of ARA and DPA than Lohmann. These findings suggest that genetic background influenced the endogenous conversion of essential fatty acids rather than their deposition into the yolk [51]. In addition, these observations are consistent with the findings of Gakhar et al. [52], indicating that the availability of dietary PUFA precursors does not necessarily translate into proportional accumulation of long-chain PUFA in eggs, likely because of differences in endogenous fatty acid metabolism.

Breed-dependent variations were observed in the estimated Δ5-, Δ6-desaturase and ELOVL-5 activities, indicating differences in the regulation of long-chain PUFA biosynthetic pathways that may be linked to genotype-dependent variation in fatty acid metabolism, as also considered by Perini et al. [53]. Hence, in the present study, while the adopted agroecological rearing system with dietary HS enrichment influenced the yolk fatty acid enrichment, genotype remained an important determinant of biologically active long-chain PUFA conversion.

The marked reduction in SFA proportions, together with the concomitant increase in DFA/OFA ratio, HPI and EFA further strengthened the functional value of eggs produced under the described field conditions. These changes were closely associated with the progressive enrichment of yolks with polyunsaturated fatty acids and the reduction of palmitic acid, one of the fatty acids most strongly associated with adverse cardiovascular outcomes [54,55,56,57]. In contrast, AI progressively declined throughout the study, reflecting a reduction of atherogenic saturated fatty acids to the overall lipid profile.

The longitudinal improvement observed in these nutritional indices is particularly relevant because they provide a more comprehensive assessment of lipid quality than individual fatty acids alone. Indeed, AI integrates the balance between fatty acids considered pro-atherogenic and those exerting protective effects on cardiovascular health, whereas DFA/OFA, HPI and EFA evaluate the relative abundance of fatty acids associated with favorable lipid metabolism and reduced cardiovascular risk [27,28,29]. Moreover, TI followed the same trend exhibited in AI and reached the lowest values at the end of the study period. TI is a key parameter for evaluating the tendency of clot formation in blood vessels; in the present study, yolks from all four breeds were characterized by a longitudinal reduction in TI values. Consequently, the simultaneous increase in DFA/OFA, HPI and EFA together with the reduction in AI and TI, indicates an overall improvement in the nutritional quality of yolk lipids of all four breeds throughout the study period.

From a human nutrition perspective, the demand for functional foods capable of providing health benefits beyond basic nutrition is increasing [58]. Eggs enriched with n-3 PUFA and characterized by favorable lipid quality indexes may represent an accessible and cost-effective dietary source of essential fatty acids, contributing to a more balanced fatty acid intake and potentially supporting cardiovascular health [59]. Therefore, the adopted agroecological rearing system with dietary HS enrichment appears to be an effective strategy to enhance the functional properties of eggs.

The linear increase in total phenolic content and antioxidant capacity of digested yolks from all four breeds represented another highly relevant result of the present study. Both TPC and TAOC progressively increased throughout the experimental period independently of breed. These findings likely reflect the combined contribution of hemp-derived bioactive compounds and pasture-derived phytochemicals. Hemp seeds contain several antioxidant molecules, including tocopherols, polyphenols, and bioactive peptides, which may contribute to reducing oxidative processes [60]. Lipophilic compounds contained in hemp seeds can be deposited in yolk, not only conditioning the total phenolic content of the final product but also the antioxidant capacity [17,61]. However, the adopted grazing system may have contributed to the observed results, as fresh pasture vegetation is a valuable source of natural antioxidants, carotenoids, and phenolic compounds [62]. Specifically, the pasture used in the present study was composed of *Lolium perenne*, *Trifolium pratense*, and *Triticum aestivum*, species that have been reported to contain appreciable concentrations of polyphenols and other antioxidant compounds, potentially contributing to the antioxidant intake of grazing animals [63,64,65,66]. Considering the adopted field conditions, future studies should focus on the characterization of lipophilic compounds to confirm their deposition in yolk.

Overall, the increased total phenolic content and antioxidant capacity of the digested yolk, together with the more favorable fatty acid profile observed following hemp seed supplementation, indicate an improvement in the nutritional characteristics of the eggs. These changes may also contribute to improving consumer health and greater oxidative stability during storage and processing [67,68], although these aspects were not directly evaluated in the present study.

Collectively, these findings support the adopted agroecological rearing system with dietary HS enrichment as an effective strategy for producing functional eggs. Nevertheless, the absence of a non-supplemented control group and the simultaneous exposure of hens to pasture prevent the independent quantification of the contribution of HS supplementation and grazing activity. In addition, independent replicate pens per breed would be required to distinguish breed effects from housing effects. A further limitation of the present exploratory field study was the lack of systematic records of individual feed intake, body weight, and production performance, which were not collected because of routine husbandry constraints. Future studies should therefore include appropriate control groups to disentangle these effects and further evaluate the oxidative stability, sensory characteristics, and consumer acceptance of eggs derived from these productions.

## 5. Conclusions

The present exploratory field study demonstrated that the adopted agroecological system characterized by a hemp seeds dietary enrichment can improve the nutritional and functional quality of eggs.

Although breed-specific differences were observed in egg quality traits and lipid metabolism, local breeds maintained satisfactory nutritional characteristics under the agroecological conditions described. These findings support the potential valorization of local genetic resources within alternative farming systems. Overall, the adopted agroecological rearing practices appear to be a promising strategy for producing value-added eggs with enhanced nutritional and functional properties.

## Figures and Tables

**Table 1:** Basal diet and HS chemical analyses.

Chemical Components, % as Fed ^1,2^	Feed	HS
Dry matter	92.75 ± 1.22	91.84 ± 1.07
Crude protein	17.01 ± 0.70	22.52 ± 0.89
Crude fat	3.38 ± 0.21	25.75 ± 0.67
Crude fiber	7.09 ± 0.31	28.05 ± 1.18
Ash	10.74 ± 0.63	4.81 ± 0.24
Fatty acids, %		
Palmitic (C16:0)	15.17 ± 0.05	7.09 ± 0.02
Palmitoleic (C16:1)	0.12 ± 0.01	0.11 ± 0.01
Stearic (C18:0)	0.22 ± 0.06	2.49 ± 0.05
Oleic (C18:1)	29.00 ± 0.07	14.89 ± 0.08
Linoleic (C18:2)	53.26 ± 0.09	54.63 ± 0.08
α-Linolenic (C18:3α)	1.24 ± 0.10	17.42 ± 0.04
γ-Linolenic (C18:3γ)	0.15 ± 0.01	1.47 ± 0.02
Arachidic (C20:0)	0.32 ± 0.01	0.74 ± 0.02
Eicosadienoic (C20:2)	0.23 ± 0.01	0.62 ± 0.01
Behenic (C22:0)	0.17 ± 0.01	0.38 ± 0.01
Lignoceric (C24:0)	0.12 ± 0.01	0.16 ± 0.01

^1^ Ingredients: maize, maize gluten, wheat byproducts, dehulled and toasted soybean meal (44% CP), calcium carbonate, dehulled sunflower meal, monocalcium phosphate, soybean oil, sodium chloride. Vitamin-mineral premix (per kg of diet): Vitamin A 8000 IU, Vitamin D3 2500 IU, Vitamin E 25 mg, Choline 350 mg, Fe 35 mg, Cu 8 mg, Mn 105 mg, Zn 42 mg, I 0.50 mg, Se 0.30 mg, DL-methionine 1470 mg, 6-phytase 600 FYT, Endo-1,4-beta-glucanase 250 TGU, Endo-1,4-beta-xylanase 560 TXU. ^2^ Chemical analyses were performed on n = 5 samples; results are indicated as mean ± standard deviation (SD).

**Table 2:** Egg physical parameters assessed throughout the study.

	Months				Fixed Effects ^1^			
Parameter	0	3	6	9	SEM	Time	Breed	Time × Breed
Shell Thickness (mm)								
MAR	0.36	0.41	0.40	0.33	0.029	<0.01 (0.47)	0.04 (0.06)	0.80 (0.04)
LIV	0.36	0.44	0.40	0.35				
MIL	0.36	0.41	0.41	0.34				
LOH	0.36	0.43	0.40	0.36				
Shape Index								
MAR	76.24	75.66	76.81	75.64	2.80	0.11 (0.04)	<0.001 (0.19)	0.31 (0.07)
LIV	74.21	74.13	75.83	74.50				
MIL	77.00	73.77	73.34	74.76				
LOH	78.83	76.59	78.21	77.72				
Whole egg weight (g)								
MAR	56.54 ^BX^	65.54 ^AW^	70.88 ^AW^	69.00 ^AW^	4.20	<0.01 (0.32)	0.20 (0.03)	<0.01 (0.23)
LIV	56.92 ^BY^	71.25 ^AW^	66.47 ^AWX^	61.95 ^BXY^				
MIL	66.83 ^AW^	70.22 ^AW^	66.11 ^AW^	64.73 ^ABW^				
LOH	61.37 ^ABY^	70.88 ^AW^	69.90 ^AW^	63.72 ^ABXY^				
Yolk (%)								
MAR	24.88 ^BW^	28.64 ^AW^	29.39 ^AW^	29.40 ^AW^	2.40	0.10 (0.04)	<0.01 (0.32)	<0.01 (0.22)
LIV	30.17 ^AW^	28.94 ^AW^	27.25 ^ABW^	29.37 ^AW^				
MIL	31.36 ^AW^	28.67 ^AW^	29.67 ^AW^	31.68 ^AW^				
LOH	25.40 ^BW^	27.35 ^AW^	25.66 ^BW^	26.13 ^BW^				
Albumen (%)								
MAR	64.48 ^AW^	61.31 ^AW^	61.04 ^BW^	61.40 ^BW^	2.40	0.71 (0.01)	<0.01 (0.27)	<0.01 (0.19)
LIV	59.38 ^BW^	60.93 ^AW^	62.84 ^ABW^	61.52 ^BW^				
MIL	59.19 ^BW^	62.09 ^AW^	60.67 ^BW^	59.33 ^BW^				
LOH	64.62 ^AW^	62.83 ^AW^	64.47 ^AW^	64.38 ^AW^				
Shell (%)								
MAR	10.64 ^W^	10.04 ^W^	9.56 ^W^	9.19 ^X^	0.72	<0.01 (0.18)	<0.05 (0.08)	0.14 (0.08)
LIV	10.45 ^W^	10.13 ^W^	9.91 ^W^	9.12 ^X^				
MIL	9.45 ^W^	9.24 ^W^	9.70 ^W^	8.98 ^X^				
LOH	9.98 ^W^	9.82 ^W^	9.87 ^W^	9.49 ^X^				
Edible (%)								
MAR	89.36 ^X^	89.96 ^X^	90.44 ^X^	90.81 ^W^	0.72	<0.01 (0.18)	<0.05 (0.08)	0.26 (0.08)
LIV	89.55 ^X^	89.87 ^X^	90.09 ^X^	90.88 ^W^				
MIL	90.55 ^X^	90.76 ^X^	90.34 ^X^	91.02 ^W^				
LOH	90.02 ^X^	90.18 ^X^	90.13 ^X^	90.52 ^W^				
Yolk/Albumen								
MAR	0.39 ^BW^	0.47 ^AW^	0.48 ^AW^	0.48 ^AW^	0.06	0.25 (0.03)	<0.01 (0.28)	<0.01 (0.20)
LIV	0.51 ^AW^	0.48 ^AW^	0.43 ^ABX^	0.48 ^AW^				
MIL	0.53 ^AW^	0.46 ^AW^	0.49 ^AW^	0.54 ^AW^				
LOH	0.39 ^BW^	0.44 ^AW^	0.40 ^BW^	0.41 ^BW^				
Yolk pigmentation								
MAR	9.13 ^BY^	12.70 ^AX^	13.90 ^AW^	12.98 ^AX^	0.76	<0.01 (0.70)	<0.01 (0.22)	<0.01 (0.31)
LIV	10.10 ^AY^	12.68 ^AW^	13.30 ^ABW^	12.08 ^BX^				
MIL	11.38 ^AY^	12.40 ^AX^	13.81 ^AW^	13.25 ^AX^				
LOH	10.57 ^AX^	12.08 ^AW^	12.68 ^BW^	11.20 ^BX^				

^1^ Values in parentheses represent partial eta squared (ηp^2^) reported as an effect size measure. Abbreviations: MAR = Marans, LIV = Livorno, MIL = Milanino, LOH = Lohmann Brown^®^. All the results are reported as mean ± standard error of the mean (SEM). Different superscript letters within the same column indicate significant differences among breeds at the same sampling time (A, B: *p* < 0.05). Different superscript letters within the same row indicate significant differences among sampling times (W, X, Y: *p* < 0.05). When the time × breed interaction was significant, temporal comparisons were performed within each breed; when the interaction was not significant, temporal comparisons were performed on the marginal means across breeds. For non-significant time × breed interactions, temporal superscripts therefore apply to all breeds (W, X, Y: *p* < 0.05).

**Table 3:** Yolk chemical composition evaluated throughout the study period.

	Months				Fixed Effects ^1^			
Parameter (%, as Fed)	0	3	6	9	SEM	Time	Breed	Time × Breed
DM								
MAR	53.08	53.08	53.07	52.39	0.82	0.49 (0.04)	<0.01 (0.54)	0.99 (0.03)
LIV	50.85	50.84	50.92	50.81				
MIL	51.29	51.28	50.89	50.91				
LOH	51.88	51.88	51.69	51.57				
CP								
MAR	17.00 ^W^	17.04 ^W^	16.56 ^W^	16.12 ^W^	0.53	<0.01 (0.22)	<0.01 (0.42)	0.67 (0.10)
LIV	16.27 ^W^	15.92 ^W^	15.76 ^W^	15.70 ^W^				
MIL	16.44 ^W^	16.03 ^W^	15.46 ^W^	15.60 ^W^				
LOH	15.70 ^W^	15.69 ^W^	15.40 ^W^	15.58 ^W^				
EE								
MAR	27.22 ^W^	28.97 ^W^	27.92 ^W^	27.31 ^W^	0.91	<0.01 (0.35)	<0.01 (0.37)	0.54 (0.10)
LIV	25.95 ^W^	27.79 ^W^	26.37 ^W^	27.13 ^W^				
MIL	27.14 ^W^	28.28 ^W^	26.74 ^W^	27.79 ^W^				
LOH	27.87 ^W^	29.75 ^W^	28.67 ^W^	28.24 ^W^				
Ash								
MAR	1.88 ^AW^	1.68 ^AW^	1.64 ^AW^	1.84 ^AW^	0.13	<0.01 (0.50)	0.16 (0.18)	<0.01 (0.29)
LIV	1.90 ^AW^	1.69 ^AX^	1.66 ^AX^	1.98 ^AW^				
MIL	1.58 ^BX^	1.66 ^AX^	1.59 ^AX^	1.97 ^AW^				
LOH	1.77 ^ABW^	1.68 ^AW^	1.34 ^BX^	1.76 ^AW^				

^1^ Values in parentheses represent partial eta squared (ηp^2^) reported as an effect size measure. Abbreviations: DM = dry matter, CP = crude protein, EE = ether extract, MAR = Marans, LIV = Livorno, MIL = Milanino, LOH = Lohmann Brown^®^. All the results are reported as mean ± standard error of the mean (SEM). Different superscript letters within the same column indicate significant differences among breeds at the same sampling time (A, B: *p* < 0.05). Different superscript letters within the same row indicate significant differences among sampling times (W, X: *p* < 0.05). When the time × breed interaction was significant, temporal comparisons were performed within each breed; when the interaction was not significant, temporal comparisons were performed on the marginal means across breeds. For non-significant time × breed interactions, temporal superscripts therefore apply to all breeds (W, X: *p* < 0.05). Values sharing at least one superscript letter are not significantly different.

**Table 4:** Cholesterol content and fatty acids proportion evaluated in yolks from all four breeds during the study.

	Months				Fixed Effects ^1^			
Parameter	0	3	6	9	SEM	Time	Breed	Time × Breed
Cholesterol (g/100 g of yolk)								
MAR	1.45	1.24	1.25	1.12	0.18	0.11 (0.09)	<0.01 (0.23)	0.32 (0.14)
LIV	1.09	1.16	1.09	1.08				
MIL	1.39	1.39	1.26	1.18				
LOH	1.03	1.16	1.22	1.05				
SFA (%)								
MAR	33.21 ^BW^	35.46 ^AW^	29.75 ^BX^	29.58 ^BX^	1.30	<0.01 (0.72)	<0.01 (0.33)	<0.05 (0.25)
LIV	35.89 ^AW^	34.97 ^AW^	31.93 ^AX^	32.37 ^AX^				
MIL	34.46 ^ABW^	32.59 ^BW^	29.83 ^ABX^	30.07 ^BX^				
LOH	32.95 ^BW^	34.50 ^ABW^	30.50 ^ABX^	30.59 ^ABX^				
MUFA (%)								
MAR	46.39 ^AW^	40.87 ^AW^	35.27 ^AX^	29.85 ^AY^	2.80	<0.01 (0.81)	<0.01 (0.36)	<0.05 (0.27)
LIV	38.09 ^BW^	39.56 ^AW^	27.75 ^BX^	25.98 ^AX^				
MIL	39.81 ^BW^	38.68 ^AW^	32.67 ^AX^	26.88 ^AX^				
LOH	38.94 ^BW^	37.38 ^AW^	34.64 ^AW^	28.89 ^AX^				
PUFA (%)								
MAR	20.39 ^Y^	23.67 ^Y^	34.98 ^X^	40.57 ^W^	3.30	<0.01 (0.85)	<0.01 (0.20)	0.07 (0.21)
LIV	26.01 ^Y^	25.47 ^Y^	40.34 ^X^	41.65 ^W^				
MIL	25.73 ^Y^	28.73 ^Y^	37.39 ^X^	43.04 ^W^				
LOH	28.11 ^Y^	28.11 ^Y^	34.86 ^X^	40.52 ^W^				
n-6 PUFA (%)								
MAR	18.52 ^BY^	20.09 ^BY^	29.79 ^BX^	34.03 ^AX^	2.50	<0.01 (0.84)	<0.01 (0.27)	<0.05 (0.25)
LIV	23.66 ^AY^	22.37 ^ABY^	34.14 ^AX^	35.28 ^AX^				
MIL	23.40 ^AY^	25.17 ^AY^	31.57 ^ABX^	35.46 ^AX^				
LOH	25.69 ^AY^	24.74 ^AY^	30.20 ^ABX^	34.09 ^AX^				
n-3 PUFA (%)								
MAR	1.86 ^Z^	3.58 ^Y^	5.19 ^X^	6.53 ^W^	0.83	<0.01 (0.85)	0.11 (0.09)	0.19 (0.17)
LIV	2.34 ^Z^	3.10 ^Y^	6.18 ^X^	6.37 ^W^				
MIL	2.33 ^Z^	3.56 ^Y^	5.82 ^X^	7.58 ^W^				
LOH	2.42 ^Z^	3.38 ^Y^	4.65 ^X^	6.43 ^W^				
n-6/n-3								
MAR	9.94 ^W^	5.72 ^X^	5.98 ^Y^	5.36 ^Y^	0.82	<0.01 (0.87)	<0.05 (0.14)	0.16 (0.18)
LIV	10.22 ^W^	7.28 ^X^	5.54 ^Y^	5.56 ^Y^				
MIL	10.07 ^W^	7.22 ^X^	5.48 ^Y^	4.69 ^Y^				
LOH	10.80 ^W^	7.39 ^X^	6.59 ^Y^	5.37 ^Y^				

^1^ Values in parentheses represent partial eta squared (ηp^2^) reported as an effect size measure. Abbreviations: SFA = saturated fatty acids, MUFA = monounsaturated fatty acids, PUFA = polyunsaturated fatty acids, n-6 PUFA = omega-6 polyunsaturated fatty acids, n-3 PUFA = omega-3 polyunsaturated fatty acids, MAR = Marans, LIV = Livorno, MIL = Milanino, LOH = Lohmann Brown^®^. All the results are reported as mean ± standard error of the mean (SEM). Different superscript letters within the same column indicate significant differences among breeds at the same sampling time (A, B: *p* < 0.05). Different superscript letters within the same row indicate significant differences among sampling times (W, X, Y, Z: *p* < 0.05). When the time × breed interaction was significant, temporal comparisons were performed within each breed; when the interaction was not significant, temporal comparisons were performed on the marginal means across breeds. For non-significant time × breed interactions, temporal superscripts therefore apply to all breeds (W, X, Y, Z: *p* < 0.05). Values sharing at least one superscript letter are not significantly different.

**Table 5:** Saturated and monounsaturated fatty acids characterized in pooled yolks during the study.

	Months				Fixed Effects ^1^			
Parameter (%)	0	3	6	9	SEM	Time	Breed	Time × Breed
Palmitic (C16:0)								
MAR	24.00 ^W^	23.26 ^X^	19.87 ^Y^	19.44 ^Y^	1.00	<0.01 (0.79)	<0.05 (0.28)	0.15 (0.18)
LIV	25.83 ^W^	23.28 ^X^	22.06 ^Y^	21.36 ^Y^				
MIL	23.78 ^W^	22.07 ^X^	20.34 ^Y^	19.46 ^Y^				
LOH	23.65 ^W^	23.65 ^X^	21.14 ^Y^	19.88 ^Y^				
Palmitoleic (C16:1)								
MAR	3.48 ^W^	2.78 ^X^	2.14 ^Y^	1.83 ^Z^	0.30	<0.01 (0.85)	<0.01 (0.32)	0.08 (0.21)
LIV	3.28 ^W^	2.53 ^X^	1.57 ^Y^	1.35 ^Z^				
MIL	3.01 ^W^	2.49 ^X^	1.99 ^Y^	1.51 ^Z^				
LOH	3.20 ^W^	2.87 ^X^	2.47 ^Y^	1.79 ^Z^				
Stearic (C18:0)								
MAR	8.43 ^CX^	10.85 ^AW^	9.18 ^BXW^	9.39 ^BW^	0.52	<0.01 (0.51)	<0.01 (0.33)	<0.01 (0.38)
LIV	9.33 ^ABX^	10.63 ^AW^	10.14 ^AXW^	10.31 ^AW^				
MIL	9.60 ^AW^	9.58 ^BW^	8.69 ^BW^	9.81 ^ABW^				
LOH	8.54 ^BCW^	9.66 ^BW^	8.67 ^BW^	9.97 ^ABW^				
Oleic (C18:1)								
MAR	42.64 ^AW^	37.53 ^AX^	32.90 ^ABY^	27.82 ^AZ^	2.50	<0.01 (0.80)	<0.01 (0.36)	<0.01 (0.29)
LIV	34.55 ^BW^	36.72 ^AW^	25.99 ^BX^	24.47 ^AX^				
MIL	36.34 ^BW^	35.90 ^AW^	30.54 ^AX^	25.19 ^AY^				
LOH	35.46 ^BW^	34.19 ^AW^	31.95 ^AW^	26.90 ^AX^				

^1^ Values in parentheses represent partial eta squared (ηp^2^) reported as an effect size measure. Abbreviations: MAR = Marans, LIV = Livorno, MIL = Milanino, LOH = Lohmann Brown^®^. All the results are reported as mean ± standard error of the mean (SEM). Different superscript letters within the same column indicate significant differences among breeds at the same sampling time (A, B, C: *p* < 0.05). Different superscript letters within the same row indicate significant differences among sampling times (W, X, Y, Z: *p* < 0.05). When the time × breed interaction was significant, temporal comparisons were performed within each breed; when the interaction was not significant, temporal comparisons were performed on the marginal means across breeds. For non-significant time × breed interactions, temporal superscripts therefore apply to all breeds (W, X, Y, Z: *p* < 0.05). Values sharing at least one superscript letter are not significantly different.

**Table 6:** Polyunsaturated fatty acids characterized in yolks from all four breeds during the study.

	Months				Fixed Effects ^1^			
Parameter (%)	0	3	6	9	SEM	Time	Breed	Time × Breed
Linoleic (C18:2)								
MAR	15.56 ^BX^	17.41 ^BX^	27.37 ^AW^	31.48 ^AW^	2.50	<0.01 (0.85)	<0.01 (0.29)	<0.05 (0.27)
LIV	21.14 ^AX^	19.79 ^ABX^	31.50 ^AW^	32.63 ^AW^				
MIL	20.63 ^AX^	22.42 ^AX^	28.96 ^AW^	32.71 ^AW^				
LOH	23.43 ^AX^	22.43 ^AX^	27.94 ^AW^	31.73 ^AW^				
α-Linolenic (C18:3α)								
MAR	0.56 ^Y^	1.54 ^Y^	3.37 ^X^	4.48 ^W^	0.20	<0.01 (0.84)	0.28 (0.04)	0.43 (0.15)
LIV	1.06 ^Y^	1.49 ^Y^	4.37 ^X^	4.53 ^W^				
MIL	0.99 ^Y^	1.46 ^Y^	3.30 ^X^	4.90 ^W^				
LOH	1.22 ^Y^	1.72 ^Y^	3.00 ^X^	4.42 ^W^				
γ-Linolenic (C18:3γ)								
MAR	0.14 ^Z^	0.17 ^Y^	0.20 ^X^	0.27 ^W^	0.03	<0.01 (0.67)	<0.01 (0.22)	0.18 (0.16)
LIV	0.16 ^Z^	0.17 ^Y^	0.27 ^X^	0.29 ^W^				
MIL	0.11 ^Z^	0.17 ^Y^	0.21 ^X^	0.27 ^W^				
LOH	0.13 ^Z^	0.17 ^Y^	0.18 ^X^	0.22 ^W^				
Arachidonic (C20:4)								
MAR	2.68 ^AW^	2.35 ^AX^	2.01 ^ABY^	2.02 ^ABY^	0.13	<0.01 (0.44)	<0.01 (0.55)	<0.01 (0.35)
LIV	2.15 ^BCW^	2.22 ^ABW^	2.09 ^ABW^	2.04 ^ABW^				
MIL	2.46 ^AW^	2.41 ^AWX^	2.22 ^AX^	2.23 ^AX^				
LOH	2.00 ^CW^	1.99 ^BW^	1.92 ^BW^	1.92 ^BW^				
Docosapentaenoic (C22:5)								
MAR	0.18 ^AW^	0.33 ^ABW^	0.31 ^AW^	0.33 ^AW^	0.01	<0.01 (0.22)	0.40 (0.05)	<0.01 (0.32)
LIV	0.21 ^AW^	0.19 ^BW^	0.26 ^AW^	0.20 ^AW^				
MIL	0.17 ^BW^	0.25 ^BW^	0.54 ^AW^	0.53 ^AW^				
LOH	0.27 ^ABW^	0.29 ^AW^	0.35 ^AW^	0.38 ^AW^				

^1^ Values in parentheses represent partial eta squared (ηp^2^) reported as an effect size measure. Abbreviations: MAR = Marans, LIV = Livorno, MIL = Milanino, LOH = Lohmann Brown^®^. All the results are reported as mean ± standard error of the mean (SEM). Different superscript letters within the same column indicate significant differences among breeds at the same sampling time (A, B, C: *p* < 0.05). Different superscript letters within the same row indicate significant differences among sampling times (W, X, Y, Z: *p* < 0.05). When the time × breed interaction was significant, temporal comparisons were performed within each breed; when the interaction was not significant, temporal comparisons were performed on the marginal means across breeds. For non-significant time × breed interactions, temporal superscripts therefore apply to all breeds (W, X, Y, Z: *p* < 0.05). Values sharing at least one superscript letter are not significantly different.

## Data Availability

The original contributions presented in the study are included in the article/Supplementary Materials, further inquiries can be directed to the corresponding author.

## Supplementary Materials

The following supporting information can be downloaded at https://www.mdpi.com/article/10.3390/foods15173095/s1, Supplementary File S1: Additional information concerning local Milanino breed; Supplementary File S2: in vitro digestion of egg yolks.

## Abbreviations

The following abbreviations are used in this manuscript:

ABTS	2,2′-Azinobis-(3-ethylbenzothiazoline-6-sulfonic acid)
AI	Atherogenic Index
AOAC	Association of Official Analytical Chemists
AOCS	American Oil Chemists’ Society
BHT	Butylated Hydroxytoluene
CF	Crude Fiber
CI	95% Confidence Interval
CP	Crude Protein
DFA	Desirable Fatty Acids
DM	Dry Matter
EE	Ether Extract
EFA	Essential Fatty Acids
ELOVL-5	Elongation of Very Long-Chain Fatty Acids Protein 5
ELOVL-6	Elongation of Very Long-Chain Fatty Acids Protein 6
FAMEs	Fatty Acid Methyl Esters
HPI	Health-Promoting Index
HS	Hemp Seed
LIV	Livorno
LOH	Lohmann Brown^®^
MAR	Marans
MIL	Milanino
MUFA	Monounsaturated Fatty Acids
NVI	Nutritional Value Index
OFA	Undesirable Fatty Acids
PUFA	Polyunsaturated Fatty Acids
SCD-16	Stearoyl-CoA Desaturase 16
SCD-18	Stearoyl-CoA Desaturase 18
SEM	Standard Error of the Mean
SFA	Saturated Fatty Acids
TAE	Tannic Acid Equivalents
TAOC	Total Antioxidant Capacity
TE	Trolox Equivalents
THC	Δ9-Tetrahydrocannabinol
TI	Thrombogenic Index
TPC	Total Phenolic Content
n-3	Omega-3 Polyunsaturated Fatty Acids
n-6	Omega-6 Polyunsaturated Fatty Acids
