# Supplementary material for: An Exploratory Field Study on Egg Quality and Yolk Functional Properties in Commercial and Local Laying Hen Breeds Fed Whole Hemp Seed Under Agroecological Conditions
Source: Foods. 2026 Aug 31;15(17):3095. doi: 10.3390/foods15173095 (PMC13565264; doi:10.3390/foods15173095)
Supplement: Supplementary file 1 [file foods-15-03095-s001.zip › Supplementary_S1.pdf]

An Exploratory Field Study on Egg Quality and Yolk Functional Properties in Commercial and Local Laying Hen Breeds Fed Whole Hemp Seed Under Agroecological Conditions

Luca Marchetti [1], Niko Gioacchino Zeni [1], Luisa Zaniboni [1,*], Carlotta Giromini [1,2], Davide Lanzoni [1,3], Paola Antonia Corsetto [4], Angela Maria Rizzo [4], Lorenzo Colombini [5], Alice Giulia Dal Borgo [5], Giuseppe Gambazza [5] and Raffaella Rebucci [1]

[1] Department of Veterinary Medicine and Animal Sciences (DIVAS), University of Milan, Via dell' Università 6, 26900 Lodi, Italy; luca.marchetti1@unimi.it (L.M.); niko.zeni@unimi.it (N.G.Z.); carlotta.giromini@unimi.it (C.G.); raffaella.rebucci@unimi.it (R.R.)
[2] Institute for Food, Nutrition and Health, University of Reading, Reading RG6 6EU, UK
[3] Department of Human Science and Quality of Life Promotion, Università Telematica San Raffaele, 00166 Rome, Italy; davide.lanzoni@uniroma5.it (D.L.)
[4] Department of Pharmacological and Biomolecular Sciences "Rodolfo Paoletti", University of Milan, Via Balzaretti 9, 20133 Milan, Italy; paola.corsetto@unimi.it (P.A.C.); angelamaria.rizzo@unimi.it (A.M.R.)
[5] Department of Cultural and Environmental Heritage, University of Milan, Via Noto 6, 20142 Milan, Italy; lorenzo.colombini@unimi.it (L.C.); alice.dalborgo@unimi.it (A.G.D.B.); giuseppe.gambazza@unimi.it (G.G.)
* Correspondence: luisa.zaniboni@unimi.it

Supplementary File S1: Additional Information Concerning Local Milanino Breed

Milanino is a synthetic chicken breed originally developed in the early 20th century in the Milan area by crossing White Valdarnese roosters with White Orpington hens, followed by selection for a distinctive phenotype [1,2]. Initially widespread in traditional family farms for both meat and egg production, the breed became extinct during the second half of the 20th century following the replacement of local breeds with highly productive commercial hybrids [3]. The breed was successfully reconstituted by the University of Milan, Department of Veterinary Medicine and Animal Sciences (DIVAS), through the replication of the original crossbreeding scheme described in historical poultry manuals and subsequent phenotypic selection [2]. The breeding stock is currently conserved in situ at the Avian Centre for the Conservation of Local Genetic Resources in Lodi. Milanino chickens are characterized by white plumage and smooth pinkish-white shanks. Adult roosters weight approximately 3.5 kg and exhibit a broad chest and a well-developed red comb, whereas hens weight 2.5–3.0 kg and are characterized by high fertility and a strong brooding instinct.

Figure S1. Picture representing Milanino Breed and its typical traits.

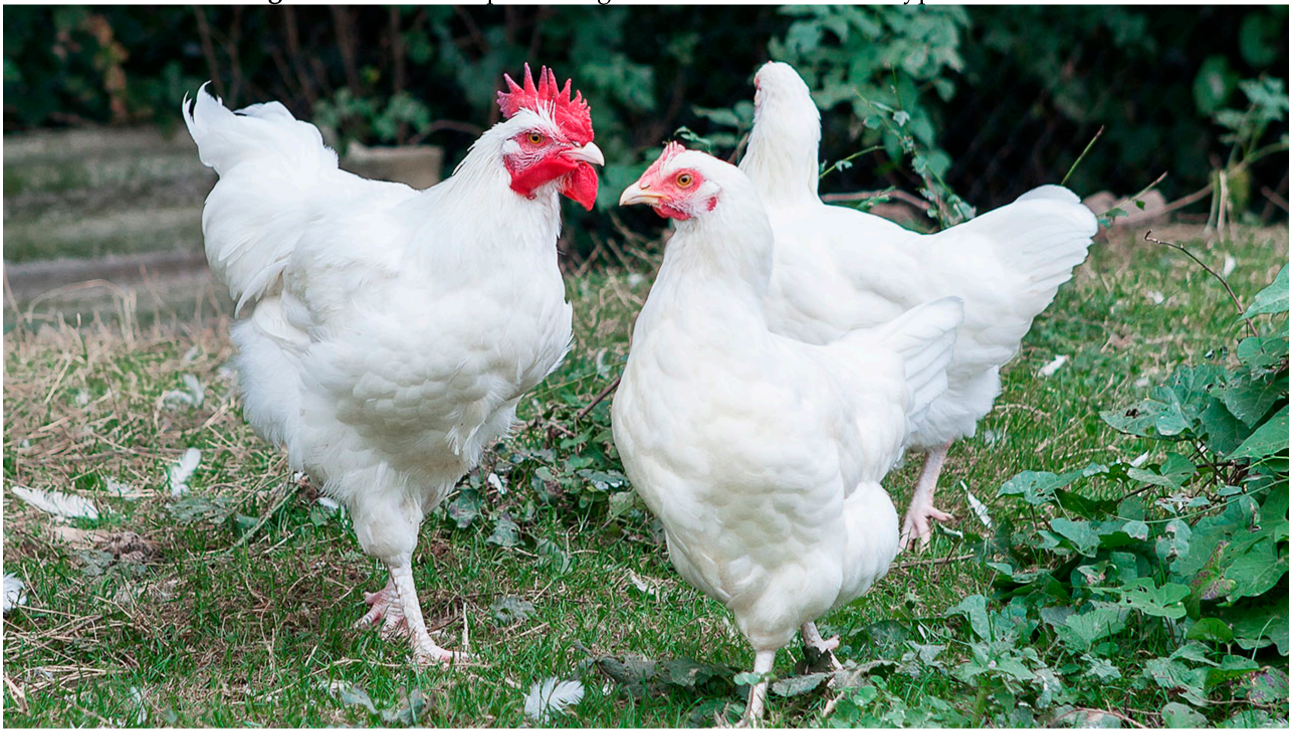

Figure S1 and the information presented therein were provided by Prof. Luisa Zaniboni, co-author of the present manuscript, who holds the copyright and ownership of the website www.pollilombardia.it and of the reported image. Permission for its use in the present manuscript was therefore granted by the copyright holder.

References

1 Cortese M. (1945) Pollicoltura familiare e industriale. Editore U. Hoepli, Milano
2 Faelli F. (1923) Animali da cortile. Editore U. Hoepli
3 Zanon A., Sabbioni A. (2001) Identificazione e salvaguardia genetica delle razze avicole italiane, Annali della Facoltà di Medicina Veterinaria di Parma, 21, 117-134.
