# Supplementary material for: An Exploratory Field Study on Egg Quality and Yolk Functional Properties in Commercial and Local Laying Hen Breeds Fed Whole Hemp Seed Under Agroecological Conditions
Source: Foods. 2026 Aug 31;15(17):3095. doi: 10.3390/foods15173095 (PMC13565264; doi:10.3390/foods15173095)
Supplement: Supplementary file 1 [file foods-15-03095-s001.zip › Supplementary_S2.pdf]

An Exploratory Field Study on Egg Quality and Yolk Functional Properties in Commercial and Local Laying Hen Breeds Fed Whole Hemp Seed Under Agroecological Conditions

Luca Marchetti [1], Niko Gioacchino Zeni [1], Luisa Zaniboni [1,*], Carlotta Giromini [1,2], Davide Lanzoni [1,3], Paola Antonia Corsetto [4], Angela Maria Rizzo [4], Lorenzo Colombini [5], Alice Giulia Dal Borgo [5], Giuseppe Gambazza [5] and Raffaella Rebucci [1]

[1] Department of Veterinary Medicine and Animal Sciences (DIVAS), University of Milan, Via dell' Università 6, 26900 Lodi, Italy; luca.marchetti1@unimi.it (L.M.); niko.zeni@unimi.it (N.G.Z.); carlotta.giromini@unimi.it (C.G.); raffaella.rebucci@unimi.it (R.R.)
[2] Institute for Food, Nutrition and Health, University of Reading, Reading RG6 6EU, UK
[3] Department of Human Science and Quality of Life Promotion, Università Telematica San Raffaele, 00166 Rome, Italy; davide.lanzoni@uniroma5.it (D.L.)
[4] Department of Pharmacological and Biomolecular Sciences "Rodolfo Paoletti", University of Milan, Via Balzaretti 9, 20133 Milan, Italy; paola.corsetto@unimi.it (P.A.C.); angelamaria.rizzo@unimi.it (A.M.R.)
[5] Department of Cultural and Environmental Heritage, University of Milan, Via Noto 6, 20142 Milan, Italy; lorenzo.colombini@unimi.it (L.C.); alice.dalborgo@unimi.it (A.G.D.B.); giuseppe.gambazza@unimi.it (G.G.)
* Correspondence: luisa.zaniboni@unimi.it

Supplementary File S2: In vitro digestion of egg yolks

For *in vitro* digestion, the Infogest protocol was applied as reported by Minekus et al [1] and Brodkorb et al [2]. Simulated digestion fluids for the oral (SOF), gastric (SGF), and intestinal (SIF) phases were prepared in agreement with Brodkorb et al [2]. For the oral phase, 2.5±0.05 g of each sample was diluted with SOF (1:1) and incubated for 2 min at 37ºC, shaking with α-amylase (75 U/mL, pH 7.0). Subsequently, the oral bolus was diluted with SGF (1:1) and pepsin (2.000 U/mL, pH 3.0) and incubated at 37ºC for 2 h under agitation. At the end of the incubation, the gastric chyme was diluted with SIF (1:1) and incubated with bile salts (10 mM, pH 7.0) and pancreatic enzymes (100 U/mL, pH 7.0) for 2 h at 37ºC, under shaking condition. Before the functional profile was evaluated, the samples were stored at -20ºC.

Total phenolic content (TPC) and antioxidant activity (ABTS) of hemp co-product, diets and eggs

For TPC, 200 µL of each sample were incubated with 1.0 mL of Folin-Ciocalteu and 0.800 µL of sodium carbonate in the dark at RT for 20 min. After incubation, the samples were read with a spectrophotometer at a wavelength of 630 nm. Data were expressed in terms of tannic acid equivalent (mg TAE/100 g). Antioxidant activity was determined using the ABTS radical cation decolorization assay according to the method of Re et al. [3] with minor modifications. Briefly, the ABTS•+ radical cation was generated by mixing 5 mL of ABTS solution (7 mM) with 88 µL of potassium persulfate ($K_2S_2O_8$, 2.45 mM). The mixture was incubated in the dark at room temperature (RT) for 16 h prior to use. The resulting ABTS•+ solution was then diluted with ethanol to obtain an absorbance of 0.700 ± 0.020 at 734 nm. For the assay, 10 µL of sample were mixed with 1.0 mL of the diluted ABTS•+ solution and incubated for 6 min at RT in the dark. Absorbance was subsequently measured at 734 nm using a spectrophotometer. Trolox was used as the reference antioxidant standard. Calibration curves were prepared using six Trolox concentrations ranging from 0 to 2000 µM. Antioxidant activity was calculated from the calibration curve and expressed as mg Trolox equivalents (TE) per 100 g of

sample.

References

1. Minekus, M.; Alminger, M.; Alvito, P.; Balance, S.; Bohn, T.; Bourlieu, C.; Carrière, F.; Boutrou, R.; Corredig, M.; Dupont, D.; Dufour, C.;… Brodkorb, A. A standardised static in vitro digestion method suitable for food - an international consensus. *Food Funct*. **2014**, 5(6), 1113-24. doi: 10.1039/c3fo60702j.

2. Brodkorb, A.; Egger, L.; Alminger, M.; Alvito, P.; Assunção, R.; Balance, S.; Bohn, T.; Bourlieu-Lacanal, C.; Boutrou, R.; Carriere, F.;…Recio, I. INFOGEST static in vitro simulation of gastrointestinal food digestion. *Nat Protoc*. **2019**, 14(4),991–1014. doi:10.1038/s41596-018-0119-1.

3. Re, R.; Pellegrini, N.; Proteggente, A.; Pannala, A.; Yang, M.; Rice-Evans, C. Antioxidant activity applying an improved ABTS radical cation depigmentationization assay. *Free Radic Biol Med*. **1999**, 26(9-10),1231-7. doi: 10.1016/s0891-5849(98)00315-3.
